# A STAT5-Smad3 dyad regulates adipogenic plasticity of visceral adipose mesenchymal stromal cells during chronic inflammation

**DOI:** 10.1038/s41536-022-00244-5

**Published:** 2022-08-31

**Authors:** Rahul Das, Jayeeta Giri, Pradyut K. Paul, Nicole Froelich, Raghavan Chinnadurai, Sara McCoy, Wade Bushman, Jacques Galipeau

**Affiliations:** 1 Department of Medicine, University of Wisconsin-Madison, Madison, WI 53705 USA; 2 Department of Urology, School of Medicine and Public Health, University of Wisconsin-Madison, Madison, WI 53705 USA; 3 Present Address: School of Medicine, Mercer University, Savannah, GA 31404 USA

**Keywords:** Mesenchymal stem cells, Mechanisms of disease, Stress signalling, Regeneration, Obesity

## Abstract

Adipogenic differentiation of visceral adipose tissue-resident multipotent mesenchymal stromal cells (VA-MSC) into adipocytes is metabolically protective. Under chronic inflammatory stress, this neoadipogenesis process is suppressed by various pro-inflammatory cytokines and growth factors. However, the underlying mechanism(s) regulating VA-MSC plasticity remains largely unexplored. Using an adipogenic differentiation screen, we identified IFNγ and TGFβ as key inhibitors of primary human VA-MSC differentiation. Further studies using human and mouse VA-MSCs and a chronic high-fat diet-fed murine model revealed that IFNγ/JAK2-activated STAT5 transcription factor is a central regulator of VA-MSC differentiation under chronic inflammatory conditions. Furthermore, our results indicate that under such conditions, IFNγ-activated STAT5 and TGFβ-activated Smad3 physically interact via Smad4. This STAT5–Smad4-Smad3 complex plays a crucial role in preventing the early adipogenic commitment of VA-MSCs by suppressing key pro-adipogenic transcription factors, including CEBPδ, CEBPα, and PPARγ. Genetic or pharmacological disruption of IFNγ-TGFβ synergy by inhibiting either STAT5 or Smad3 rescued adipogenesis under chronic inflammatory stress. Overall, our study delineates a central mechanism of MSC plasticity regulation by the convergence of multiple inflammatory signaling pathways.

## Introduction

White adipose tissue (WAT) is central to maintaining systemic energy homeostasis^1^. During periods of caloric excess, WAT absorbs and stores excess circulatory nutrients as triglycerides and mobilize them during caloric demand. In addition, adipocytes secrete various hormones and cytokines, collectively known as “adipokines” that co-ordinate metabolism in different tissues^2^. Adipocyte dysfunction, particularly related to visceral fat depots, is linked to metabolic disorders including insulin resistance, metabolic syndrome, and type 2 diabetes^3–5^.

Short-term excess caloric consumption causes expansion of visceral and intra-abdominal WAT through increases in adipocyte size (hypertrophy) and number (hyperplasia)^6,7^. Hyperplasia occurs through neoadipogenesis in a two-step differentiation process^8,9^; (a) commitment, where adipose tissue-resident multipotent mesenchymal stromal cells (MSCs) convert into preadipocytes by expressing early adipogenic transcription factors, and (b) terminal differentiation, where preadipocytes convert into mature adipocytes by accumulating lipid compounds, mostly triglycerides. However, under chronic inflammatory conditions such as prolonged high-caloric diet consumption, adipose tissue is infiltrated by pro-inflammatory immunocytes, including macrophages, T cells, etc. These immunocytes secrete potent cytokines, interleukins and chemokines that impair normal adipose tissue function and prevent WAT hyperplasia. As a result, adipocytes grow mostly by hypertrophy, leading to adipocyte dysfunction, further immune-infiltration, and inflammatory response that cause adipocyte death. At the same time, lack of hyperplasia prevents functional adipose regeneration, which contributes to various metabolic disorders^3,10,11^. Similarly, loss of functional adipose tissue and proper adipocyte function (such as adipokine secretion) is intimately linked with “wasting syndromes” that accompany chronic diseases, including cancer cachexia, chronic kidney disease, thyroid disease, and chronic liver failure^12–14^. Wasting syndromes causes systemic depletion of muscle and adipose tissue mass, diminished metabolic and physiological activities, and are linked with reduced quality of life and increased mortality. Adipogenic regeneration by therapeutic means may provide beneficial outcome in such cases.

Molecular regulation of terminal adipogenic differentiation has been widely studied using mouse embryonic fibroblasts, committed primary human/ mouse preadipocytes, and mouse preadipocyte cell line 3T3-L1^15,16^. Although fundamental insights were gained from such studies, these cell types phenotypically vary wildly^17^, and do not represent the multipotent nature of primary adipose MSCs^18^. How physiologically relevant chronic inflammatory conditions regulate the adipogenic plasticity of visceral MSCs remain largely unexplored. Therefore, we sought to identify and characterize evolutionarily conserved molecular pathway(s) that control the differential potential of adipogenic MSCs.

Using an in vitro differentiation screen with pro-inflammatory cytokines and growth factors, we identified IFNγ and TGFβ as strong inhibitors of primary human visceral adipose MSC (VA-MSC) early adipogenic commitment. A long-term high-fat diet-fed mouse model revealed that IFNγ is essential for regulating visceral adipogenesis under meta-inflammatory conditions. IFNγ activated several STAT transcription factors in VA-MSCs, among which STAT5 was found to be essential for inhibiting adipogenesis. Further molecular characterization revealed that IFNγ-activated STAT5 and TGFβ-activated Smad3 physically interact via Smad4. This interaction is essential for preventing adipogenic differentiation. Pharmacological disruption of the STAT5–Smad3 dyad salvaged adipogenesis by allowing expression of key pro-adipogenic transcription factors, including C/EBPδ and PPARγ. As an aggregate, these data show that a chronic inflammation-driven pathologic positive feedback loop, composed of reciprocal activation of STAT5 and Smad3 proteins, is the central molecular mechanism that prevents adipose hyperplasia in a physiologically relevant setting. Our findings, therefore, provide a druggable approach by inducing adipogenesis via targeting IFNγ and TGFβ pathways in the context of metabolic dysfunction and wasting syndrome with underlying chronic inflammation.

## Results

### IFNγ and TGFβ are major inhibitors of VA-MSC differentiation under chronic inflammatory conditions

**Fig. 1:**
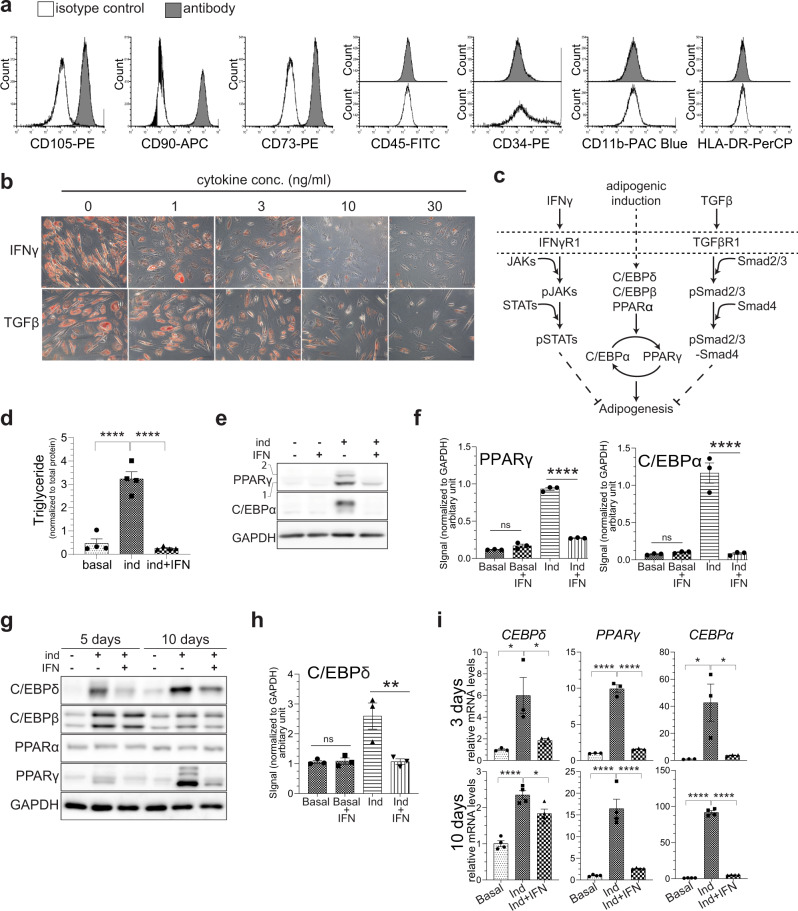
IFNγ is a potent inhibitor of adipogenesis from human visceral adipose-derived MSCs. **a** Phenotypic characterization of human VA-MSC by flow cytometry. Representative figure (*N* = 3) showing MSC-specific marker expression (CD105^+^CD73^+^CD90^+^CD45^−^CD34^−^CD11b^−^HLADR^−^) in one hVA-MSC. **b** Representative (*N* = 3) phase-contrast microscopy image of Oil Red-stained hVA-MSC after 14 days of adipogenic differentiation with/out human IFNγ and TGFβ. Media, along with IFNγ or TGFβ was changed every 48 h. **c** Schematic diagram depicting canonical IFNγ and TGFβ signaling pathways and adipogenesis cascade. **d** Colorimetric triglyceride assay of hVA-MSC extract after 14 days of adipogenic induction with/out human IFNγ (10 ng/ml). Triglyceride levels were normalized to that of total protein (*N* = 4). **e**, **f** Representative western blot analysis (**e**) and blot quantification (**f**, *N* = 3) of hVA-MSC after 14 days of adipogenic stimulation with/out IFNγ (10 ng/ml). **g** Representative western blot analysis of hVA-MSC after 5 or 10 days of adipogenic differentiation with/out IFNγ (10 ng/ml). **h** Quantification of C/EBPδ band (*N* = 3) after 5 days of adipogenic induction. **i** qRT-PCR quantification of cDNA prepared from hVA-MSCs after 3 or 10 days of adipogenic induction with/out IFNγ (10 ng/ml). Transcript levels were normalized to that of *GAPDH* (*N* = 3/4). Ind-adipogenic induction. Normal cell culture media (Ind^−^IFN^−^) treated cells were used as the basal condition. Error bars represent mean ± SEM. * indicates statistical significance (*P < 0.05; **P < 0.005, ***P < 0.0005, ****P < 0.00005) of Tukey’s multiple comparisons test post one-way ANOVA analysis.

Under chronic inflammatory stress, visceral WAT infiltrating macrophages, T cells, as well as stressed adipocytes themselves secrete various pro-inflammatory factors that inhibit adipogenesis. Modulatory effects of many pro-inflammatory cytokines on terminal adipogenic differentiation has been studied using various committed preadipocytes, such as 3T3-L1^16^. However, how these factors affect adipogenic potential of VA-MSCs remain largely unknown. To study VA-MSC plasticity under chronic inflammatory conditions, we first collected intra-abdominal visceral fat samples from healthy human donors undergoing elective abdominal surgery. Stromal vascular fractions were isolated from these tissue samples and then cultured ex vivo in the presence of human platelet lysate. Next, we determined whether these cells express classical MSC-specific surface proteins^19–22^. Using flow cytometry, we show that the cell-surface marker phenotype of these cells are consistent with the bona fide MSCs, i.e., CD105^+^CD73^+^CD90^+^CD45^−^CD34^−^CD11b^−^HLADR^−^ (Fig. 1a). Next, adipogenesis was induced in hVA-MSCs by incubating cells in an adipogenic cocktail for 14 days with media change at regular intervals. Cells were concomitantly treated with a dose range of individual cytokines or growth factors (total 14 cytokines) over this period. Then the degree of adipogenesis was assessed by Oil Red staining (Supplementary Fig. 1). Many of the cytokines previously shown to inhibit adipogenesis in various preadipocyte cells, such as TNFα^23^, IL-1β^24^, IL6^25^, and IL15^26^ were unable to inhibit adipogenesis in hVA-MSC. Among all the factors tested, only IFNγ (a type II interferon) and TGFβ (a multifunctional cytokine/growth factor) exhibited suppression of adipogenesis in a dose-dependent manner (Fig. 1b and Supplementary Fig. 1). As shown in Fig. 1c schematic, these two cytokines work through completely different molecular mechanisms; IFNγ through JAK-STAT signaling and TGFβ through Smad2/3 signaling. Therefore, we aimed to test the individual and combinatorial contribution of these pathaways on hVA-MSCs differentiation.

### IFNγ prevents VA-MSC adipogenic commitment by suppressing C/EBPδ and PPARγ

We first studied the role of IFNγ in VA-MSC differentiation. IFNγ signaling occurs through JAK-STAT, a highly conserved signaling pathway^27^. IFNγ is a central mediator of immunity^28^, however, its effects on VA-MSC differentiation have not been investigated. As 10 ng/ml IFNγ was sufficient to completely prevent lipid droplet accumulation in adipogenic screen, we used this concentration hereafter. Triglyceride assays using multiple (*N* = 4) donor-derived hVA-MSCs treated with adipogenic cocktail for 14 days with/out exogenous IFNγ showed that IFNγ treatment potently prevents adipogenesis (Fig. 1d).

To determine the specific mechanism of adipogenic inhibition by IFNγ (i.e., commitment or terminal differentiation), we treated VA-MSC with adipogenic cocktail as before. After 10 days of treatment, western blots were performed using antibodies against two transcription factors essential for adipogenesis, namely PPARγ (the central regulator of adipogenesis) and C/EBPα (Fig. 1c schematic and Fig. 1e). IFNγ treatment diminished protein levels of both PPARγ and C/EBPα in VA-MSCs. Further quantification of these proteins from three distinct MSC donors confirmed the inhibitory role of IFNγ (Fig. 1f). These data show that IFNγ predominantly impacts VA-MSC adipogenic commitment by preventing the buildup of PPARγ.

We next investigated the effects of IFNγ on key pre- PPARγ adipogenic transcription factors, such as C/EBPδ, PPARα and C/EBPβ (Fig. 1c schematic), that are required for the initial upregulation of PPARγ and C/EBPα expression^29–31^, after which PPARγ and C/EBPα bolster each other. VA-MSCs were treated for 5 days or 10 days with adipogenic media and IFNγ. Next, western blot analyses of cell lysates were performed using the antibodies against the transcription factors mentioned. C/EBPδ protein was detectable in hVA-MSC at day 5 of adipogenic stimulation and its levels increased by day 10 (Fig. 1g). IFNγ inhibited this C/EBPδ expression at both early and late stages of differentiation. The major function of C/EBPδ is to promote initial PPARγ gene expression, which explains observed reduction PPARγ protein expression in IFNγ-treated Va-MSCs at both early and late stages (Fig. 1g). However, IFNγ did not affect expression of other early transcription factors. Further protein-level quantification of C/EBPδ from three distinct MSC donors confirmed this inhibitory role of IFNγ on VA-MSC adipogenic commitment through C/EBPδ (Fig. 1h).

IFNγ-activated STAT transcription factors may mediate transcriptional suppression of *C/EBPδ, PPARγ*, and *C/EBPα* genes, resulting in observed reduction in protein levels. To test this, we induced adipogenesis as before in hVA-MSC and collected mRNA after 3 or 10 days, i.e., pre and post-commitment, from these cells. Then we measured transcript levels of *C/EBPδ*, *PPARγ* and *C/EBPα* (Fig. 1i). Initial *C/EBPδ* upregulation at day 3 was fully suppressed by IFNγ treatment. By day 10, the expression of C/EBPδ was reduced compared to day 3, and was partially (but significantly) suppressed by IFNγ. For *PPARγ* and *C/EBPα* genes, the degree of transcriptional upregulation increased at day 10 compared to day 3 during normal adipogenic stimulation. IFNγ completely inhibited both *PPARγ* and *C/EBPα* mRNA expression at both time points.

Collectively, these data show that pro-inflammatory cytokine IFNγ is a potent inhibitor of hVA-MSC differentiation, and IFNγ inhibits adipogenic commitment by suppressing gene and protein-level expression of C/EBPδ and PPARγ.

### Chronic IFNγ exposure activates a subset of JAK/STAT proteins in VA-MSCs

**Fig. 2:**
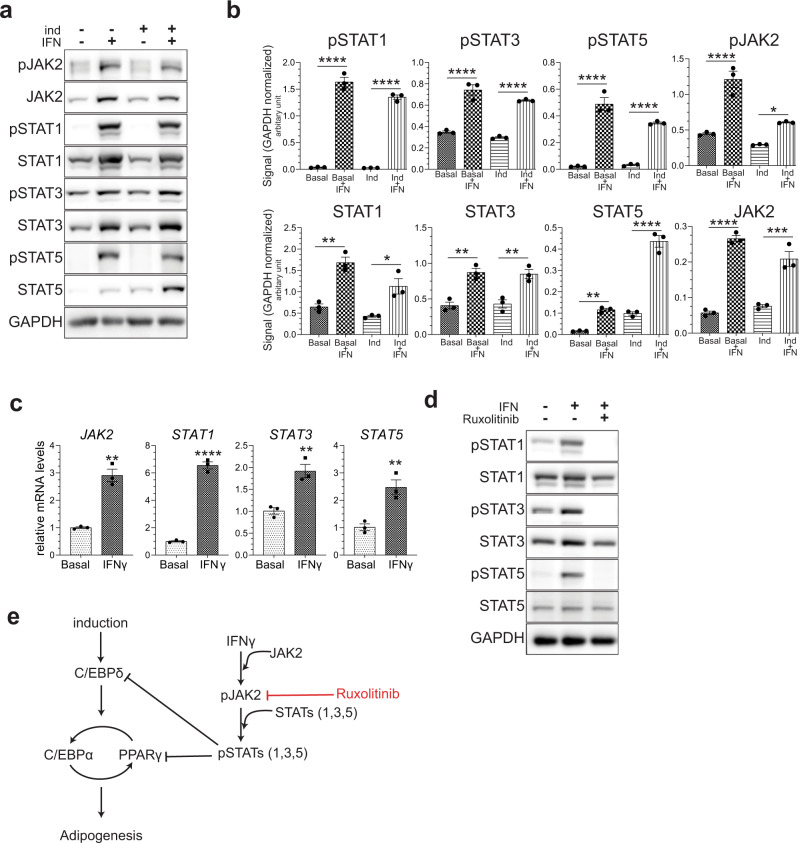
Key adipogenic transcription factors are inhibited by chronic IFNγ-induced activation of selective JAK/STAT proteins. **a**, **b** Representative western blot (**a**) and blot quantification (**b**, *N* = 3) of hVA-MSCs after 10 days of adipogenic stimulation with or without IFNγ (10 ng/ml). **c** qRT-PCR of cDNA prepared from hVA-MSCs cells after 10 days of treatment with/out IFNγ (10 ng/ml). Transcript levels were normalized to that of *GAPDH* (*N* = 3). **d** Representative hVA-MSC western blot (*N* = 2) after 12 h incubation with/out IFNγ (10 ng/ml) and/or JAK inhibitor Ruxolitinib (Ruxo) at 5 µM. **e** Schematic diagram representing the effects of IFNγ-activated JAK-STAT pathway on adipogenic commitment. Ind-adipogenic induction. Normal cell culture media (Ind^−^IFN^−^) treated cells were used as the basal condition. Error bars represent mean ± SEM. * indicates statistical significance (*P < 0.05; **P < 0.005, ****P < 0.00005) of Tukey’s test post one-way ANOVA.

There exist six STAT transcription factors^32^ that display a wide range of expression pattern and activity depending on the tissue type, developmental stage, and immunological status. How chronic IFNγ exposure affects these STAT proteins (except for STAT1) has not been interrogated. To determine the effects of long-term IFNγ exposure on JAK-STAT pathway activation, we treated hVA-MSCs with IFNγ for 10 days with or without adipogenic stimulation. Significantly increased levels of activated (i.e., tyrosine-phosphorylated) JAK2, STAT1, STAT3, and STAT5 proteins, irrespective of adipogenic induction, could be observed under such conditions (Fig. 2a). Interestingly, not only phospho-proteins but also total protein levels of JAK2, STAT1, STAT3, and STAT5 were significantly increased upon chronic IFNγ treatment (Fig. 2a), as confirmed by band quantification from multiple donors (*N* = 3, Fig. 2b). qRT-PCR analyses of mRNA isolated from hVA-MSCs treated with IFNγ for 10 days indicates that chronic IFNγ exposure indeed causes transcriptional upregulation of *JAK2*, *STAT1*, *STAT3*, and *STAT5* genes in hVA-MSCs (Fig. 2c), which results in increased protein accumulation seen in Fig. 2b. This is distinct from the reported canonical IFNγ signaling described so far where IFNγ treatment only increased phospho-STAT proteins (mostly pSTAT1) without altering total STAT protein or transcript levels^33,34^. We confirmed that short-term IFNγ treatment does not stimulate total STAT protein upregulation by treating hVA-MSCs with IFNγ for a relatively short period of time (12 h). (Fig. 2d). Under such conditions, although upregulation of pSTAT1/3/5 proteins did occur in a JAK2-dependent manner (as it could be inhibited by application of pan JAK inhibitor Ruxolitinib^35^), change in total STAT protein levels did not occur.

These results show that chronic IFNγ treatment causes transcriptional upregulation and activation of a selective subset of STAT proteins in VA-MSCs (as represented in Fig. 2e schematic), and these activated STAT proteins may play critical role in VA-MSC adipogenic differentiation.

### STAT5 is the major mediator of IFNγ’s anti-adipogenic effects

Our results so far indicated that STAT5, STAT3, and STAT1 transcription factors comprise strong candidates for regulating MSC adipogenic plasticity under chronic inflammatory stress. So, we next aimed at teasing out the function of these proteins in adipogenic inhibition.

**Fig. 3:**
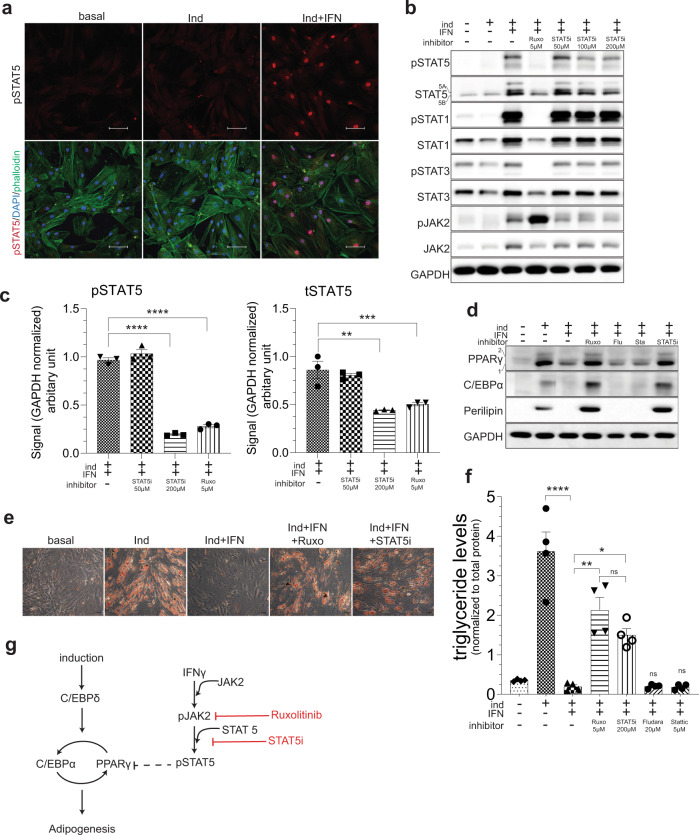
STAT5 is the major mediator of IFNγ’s anti-adipogenic action. **a** Confocal Z projection of hVA-MSC stained with pSTAT5 antibody after 10 days of adipogenic stimulation with/out IFNγ (10 ng/ml). FITC-Phalloidin (green) and DAPI (blue) were used for marking the cytoskeleton and nucleus, respectively. An image from one representative donor (*N* = 3) is shown. **b** Western blot analysis of hVA-MSCs after 10 days of adipogenic induction with/out human IFNγ (10 ng/ml) along with the inhibitors mentioned. Ruxo-Ruxolitinib. **c** Quantification of pSTAT5 and tSTAT5 bands from western blot analysis of hVA-MSCs after 10 days of adipogenic induction with IFNγ (10 ng/ml), STAT5i or Ruxolitinib at the concentrations mentioned; *N* = 3. **d** Representative phase-contrast microscopy image of Oil Red-stained hVA-MSCs (*N* = 3) after 14 days of adipogenic induction with/out IFNγ (10 ng/ml) with inhibitors mentioned (STAT5i—200 µM, Ruxolitinib—5 µM). **e** Representative western blot analysis (*N* = 3) of hVA-MSCs after 10 days of adipogenic induction with/out human IFNγ (10 ng/ml) along with the inhibitors mentioned (STAT5i—200 µM, Ruxolitinib—5 µM, Flu—fludarabine; 20 µM, Sta-stattic; 5 µM). **f** Colorimetric triglyceride assay of hVA-MSC extract after 14 days of adipogenic induction with/out human IFNγ (10 ng/ml) and inhibitor concentrations as above. Triglyceride levels were normalized to total cellular protein concentration; *N* = 4. **g** Schematic diagram representing the effects of IFNγ-activated STAT5 mediated inhibition of adipogenesis. Ind-adipogenic induction. Normal cell culture media- (Ind^−^IFN^−^) treated cells were used as the basal condition. For western blots, GAPDH was used as a loading control. Scale bar: 100 µm. Error bars represent mean ± SEM. * indicates statistical significance (*P < 0.05; **P < 0.005; ***P < 0.0005; ****P < 0.00005) of Dunnet’s test (**c**) or Tukey’s test (**f**) post one-way ANOVA.

STAT5 protein exist as two isoforms, namely STAT5A and STAT5B, and are activated by many cytokines, interleukins, and growth factors; however, regulation of STAT5 activity by IFNγ has not been investigated yet. Tyrosine-phosphorylated, active STAT5 could not be detected by immunofluorescence imaging in hVA-MSC under basal or adipogenic stimulation conditions. However, 10 days of continuous IFNγ treatment caused prominent nuclear localization of pSTAT5, indicating that IFNγ-JAK2-activated STAT5 translocate to the nucleus to regulate target gene transcription in hVA-MSCs (Fig. 3a). To interrogate the specific roles of STAT5 in adipogenic inhibition, we employed a small-molecule STAT5 inhibitor, namely STAT5i^36^. VA-MSCs were treated with adipogenic cocktail with or without IFNγ for 10 days. STAT5i was added at various doses (ranging from 50 to 200 µM) in a subset of cells. As a positive control, pan JAK2/1 inhibitor Ruxolitinib was used. Media, along with IFNγ and inhibitors was changed every 48 h. After 10 days of such treatment, western blots were performed using antibodies against JAK-STAT pathway members. As shown in Fig. 3b, STAT5i, in a dose-dependent manner, inhibited chronic IFNγ induced increases in pSTAT5 as well as total STAT5 protein levels without affecting other STAT proteins. In contrast, Ruxolitinib inhibited all STAT proteins. Quantification of pSTAT5 and total STAT5 proteins from multiple donor VA-MSCs (*N* = 3) showed that STAT5i mediated inhibition of IFNγ stimulated STAT5 is statistically significant (Fig. 3c). At a molecular level, STAT5i treatment (similar to Ruxolitinib) caused de-suppression of key adipogenic transcription factors, including PPARγ, C/EBPα, and downstream Perilipin protein expression (Fig. 3d). Phenotypically, STAT5i treatment resulted in the restoration of adipogenesis in hVA-MSCs, despite continuous presence of IFNγ, as revealed by Oil Red staining after 14 days of treatment (Fig. 3e). These effects of STAT5i is similar to that of Ruxolitinib treatment, indicating that STAT5 is the major effector of IFNγ action in VA-MSC. Triglyceride assays using multiple donor-derived hVA-MSCs validated the adipogenic restoration ability of STAT5i (as well as Ruxolitinib) under chronic IFNγ treatment conditions (Fig. 3f).

JAK-activated STAT transcription factors are known to hetero-dimerize with other STAT proteins depending on the nature of the relevant signaling pathway(s), the relative abundance of specific STAT proteins, and their activation status^37^. It is possible that STAT1/STAT3 form heterodimer with STAT5 in VA-MSCs upon chronic IFNγ exposure, which may influence adipogenic differentiation. Therefore, we examined whether STAT1 or STAT3 participate in adipogenesis modulation in hVA-MSC. pSTAT1 could only be detected under adipogenic stimulation with chronic IFNγ treatment, where it showed prominent, mostly nuclear localization (Supplementary Fig. 2a). In contrast, pSTAT3 showed nucleo-cytoplasmic localization under all conditions (Supplementary Fig. 2b). Next, we employed small-molecule inhibitors of STAT1 (Fludarabine)^38^ and STAT3 (Stattic)^39^ to determine whether IFNγ stimulated STAT1 or STAT3 inhibits VA-MSC adipogenic differentiation. Fludarabine and stattic inhibited STAT1 or STAT3 activation, respectively, in a dose-dependent manner (Supplementary Fig. 2c, d); however, these inhibitors failed to rescue IFNγ imposed differentiation inhibition (Supplementary Fig. 2e, f). Mechanistically, inhibition of STAT1 or STAT3 did not de-repress IFNγ-inhibited PPARγ and CEBPα protein expression (Fig. 3e). We further confirmed these phenotypes by measuring triglyceride levels in multiple donor-derived hVA-MSCs (Fig. 3f). Of note, fludarabine is known to exert non STAT1 specific effects. Therefore, we validated the specific inhibition of STAT1 activity by knocking down *STAT1* in hVA-MSC using DSiRNA (Supplementary Fig. 2g, h). *STAT1* knockdown caused downregulation of STAT1 protein levels (Supplementary Fig. 2g) but was unable to reverse IFNγ-induced inhibition of adipogenesis (Supplementary Fig. 2h).

Collectively, these data show that although chronic IFNγ exposure activates multiple STAT transcription factors, STAT5 but not STAT1 or STAT3 is the major modulator of IFNγ induced inhibition of VA-MSC differentiation. This centrality of STAT5 in IFNγ-inhibited adipogenesis is shown in Fig. 3g schematic.

### TGFβ/Smad3 signaling is required for the IFNγ-mediated suppression of adipogenesis

In addition to IFNγ, the adipogenesis screen identified TGFβ as another negative regulator of VA-MSC differentiation (Fig. 1a and Supplementary Fig. 1). Hypertrophic adipocytes and immune-infiltrating cells are known to secrete TGFβ that further contributes to metabolic dysfunction^40,41^. TGFβ signaling involves serine/threonine phosphorylation-activation of Smad3 and Smad2 transcription factors by cell-surface TGFβ-R1 (TGBR1) receptor^42^. Active Smad2/3 binds to co-Smad protein Smad4. Then the Smad2/3- Smad4 complex translocate to the nucleus in order to regulate target gene expression. TGFβ signaling, particularly Smad3, was shown to inhibit adipogenesis in mouse^43^. However, the regulation and natural function of this pathway during VA-MSC differentiation remain unexplored.

**Fig. 4:**
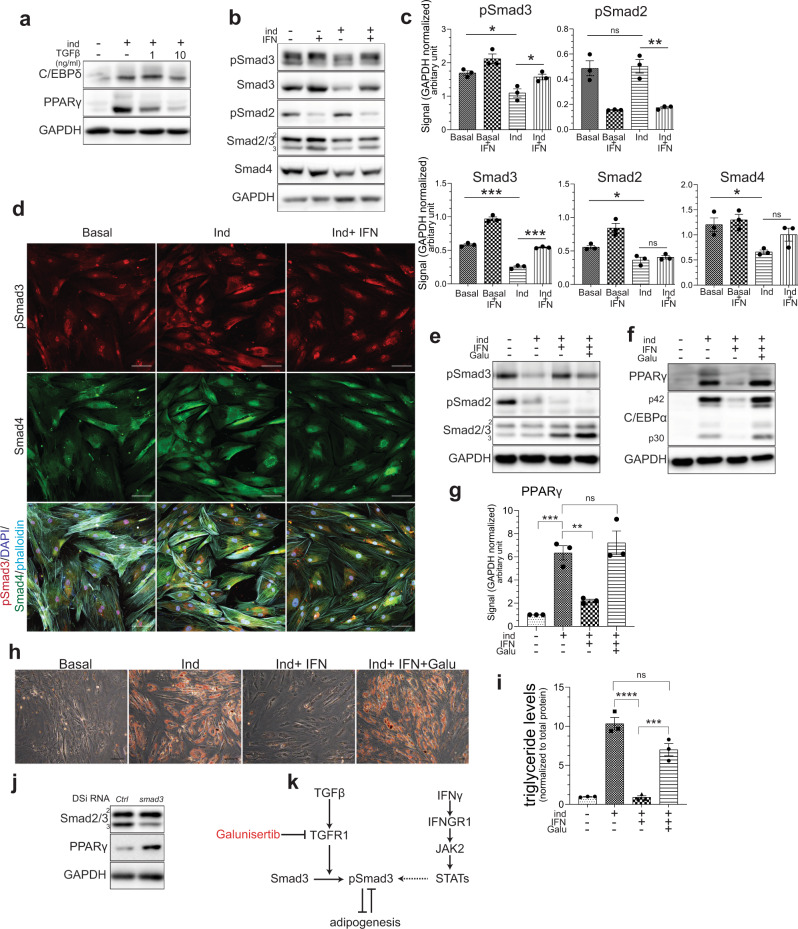
IFNγ prevents adipogenic downregulation of Smad3 to suppress VA-MSC differentiation. **a** Representative western blot analysis (*N* = 2) of hVA-MSC after 7 days of adipogenic stimulation with or without TGFβ (1 or 10 ng/ml). **b**, **c** representative western blot (**b**) and blot quantification of TGFβ pathway mediators (**c**, *N* = 3) of hVA-MSC after 10 days of adipogenic induction with/out IFNγ (10 ng/ml). **d** Confocal Z projection of hVA-MSC stained with pSmad3 and Smad4 antibodies after 10 days of adipogenic stimulation with/out IFNγ (10 ng/ml). Far red-Phalloidin (cyan) and DAPI (blue) was used for marking the cytoskeleton and nucleus, respectively. An image from one representative donor (*N* = 3) is shown. **e** Representative western blot analysis of hVA-MSC (*N* = 2) after 10 days of adipogenic induction with/out IFNγ (10 ng/ml). Galu- galunisertib, used at 20 µM. **f**, **g** Representative western blot analysis (**f**) and PPARγ band quantification (*N* = 3) of hVA-MSC (**g**) after 10 days of adipogenic induction with/out IFNγ (10 ng/ml). Galunisertib was used at 20 µM. **h** Representative phase-contrast microscopy (*N* = 3) of Oil Red-stained hVA-MSC after 14 days of adipogenic induction with/out IFNγ (10 ng/ml). Galunisertib was used at 20 µM. **i** Colorimetric triglyceride assay of hVA-MSC (*N* = 3) extracts after 14 days of adipogenic induction with/out human IFNγ (10 ng/ml) and/or Galunisertib (20 µM). Triglyceride levels were normalized to total cellular protein concentration. **j** Representative (*N* = 2) western blot analysis of DSiRNA mediated *Smad3* knocked down hVA-MSCs after 7 days of adipogenic stimulation with IFNγ (10 ng/ml). **k** Schematic diagram representing TGFβ- Smad3 and IFNγ signaling cooperativity in preventing adipogenic differentiation under chronic inflammatory conditions. Ind-adipogenic induction. Normal cell culture media (Ind^−^IFN^−^) treated cells were used as the basal condition. For western blots, GAPDH was used as loading control. Scale bar: 100 µm. Error bars represent mean ± SEM. * indicates statistical significance (*P < 0.05; **P < 0.005; ***P < 0.0005; ****P < 0.00005) of Dunnet’s test (**c**) or Tukey’s test (**g**, **i**) post one-way ANOVA.

We induced adipogenesis in hVA-MSC as before in the presence of TGFβ (1–10 ng/ml) and performed western blot analysis on cell lysates after 10 days of adipogenic stimulation (Fig. 4a). Chronic TGFβ treatment inhibited hVA-MSC adipogenic differentiation by suppressing C/EBPδ, and consequently PPARγ, in a dose-dependent manner (Fig. 4a). These effects are similar in nature to that of IFNγ in hVA-MSCs. Therefore, we sought to determine the involvement of TGFβ pathway in hVA-MSCs and cross-regulation (if any) of IFNγ and TGFβ pathways during adipogenic inhibition under chronic inflammatory conditions. For this, hVA-MSCs were adipogenically induced as before in the presence of IFNγ (10 ng/ml) and performed western blot analysis on cell lysates after 10 days of adipogenic stimulation using antibodies against phospho and total Smad proteins (Fig. 4b; quantification of bands from three different donors is shown in Fig. 4c). Adipogenic stimulation caused a general downregulation of all Smad proteins. Chronic IFNγ exposure during adipogenic stimulation specifically de-repressed phosphorylated, active pSmad3 as well as total Smad3 protein levels (Fig. 4b, c). In stark contrast to pSmad3, pSmad2 levels were significantly downregulated by IFNγ, indicating that Smad3 (but not Smad2) is a mechanistic component of chronic IFNγ signaling in hVA-MSCs. These results also indicate that pSmad3 downregulation is associated with the natural progression of adipogenic cascade, and Smad3 constitutes a crucial node in chronic inflammatory signaling pathways that inhibits adipogenic differentiation.

To determine the subcellular localization (as asurrogate for transcriptional function) of pSmad3/Smad4 proteins under various adipogenic conditions, we induced differentiation in hVA-MSC with or without IFNγ for 10 days and performed Immunofluorescence imaging on these cells. The results showed that adipogenic stimulation caused downregulation of nuclear-localized pSmad3, whereas concomitant chronic IFNγ exposure resulted in prominent nuclear localization of both pSmad3 and Smad4 (Fig. 4d). These data indicate that IFNγ driven inflammatory microenvironment activates psmad3-Smad4 complex, which may, in turn, inhibit VA-MSC differentiation. In agreement, a small-molecule inhibitor of TGFBRI kinase activity, namely Galunisertib^44^ prevented IFNγ mediated pSmad3 upregulation under adipogenic stimulatory conditions, as revealed by western blot (Fig. 4e). Consequently, Galunisertib treatment was able to rescue the protein-level expressions of key adipogenic transcription factors in chronic IFNγ exposed hVA-MSC (Fig. 3f). Quantification of the central adipogenic regulator PPARγ levels from multiple donors (*N* = 3) confirmed that Galunisertib can indeed de-repress IFNγ inhibited PPARγ expression (Fig. 4g), further showing the inter-twined nature of the IFNγ-TGFβ signalings in VA-MSCs. Phenotypically, Galunisertib treatment allowed normal adipogenic differentiation of hVA-MSC (as revealed by Oil Red imaging) despite the continuous presence of IFNγ (Fig. 4h). Triglyceride quantification of multiple donor-derived hVA-MSCs (*N* = 3), treated with adipogenic cocktail for 14 days with or without IFNγ and Galunisertib, showed that suppression of IFNγ induced Smad3 activation leads to adipocyte regeneration (Fig. 4i). To further validate the central role of Smad3 in this process, we knocked down *Smad3* in hVA-MSCs using DSiRNA. These cells were then treated with adipogenic cocktail with IFNγ. After 7 days, lysates were collected and accessed for PPARγ protein expression by western blot. DSiRNA knockdown specifically reduced Smad3 protein levels without affecting Smad2 and caused de-repression of IFNγ inhibited PPARγ protein expression (Fig. 4j).

Taken together, these data show that TGFβ activated Smad3 play a central role in IFNγ’s inhibitory effect on VA-MSC differentiation, and IFNγ & TGFβ signaling pathways co-ordinate under chronic inflammatory conditions to restrict VA-MSC plasticity (as shown in Fig. 4k diagram).

### STAT5–Smad3 cooperativity through Smad4 constitutes the functional node of the chronic IFNγ-TGFβ signaling crosstalk

**Fig. 5:**
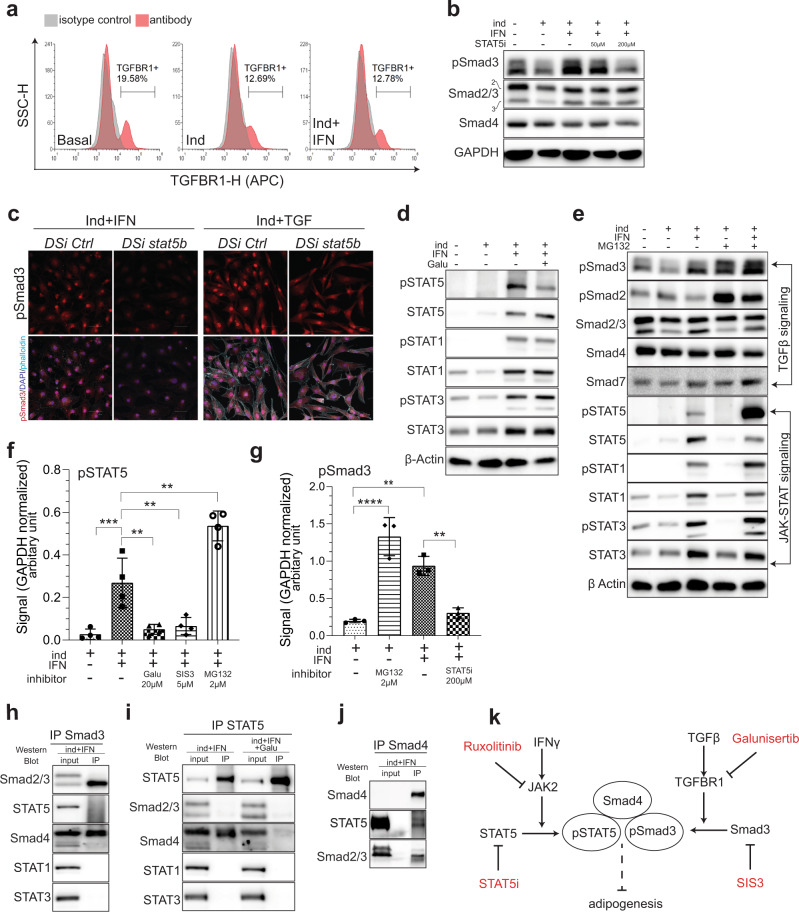
Reciprocal Smad3 and STAT5 activation constitute a central node of chronic IFNγ–TGFβ signaling synergy. **a** Representative flow cytometry analysis (*N* = 3) of hVA-MSC after 10 days of adipogenic induction with/out IFNγ (10 ng/ml). $\cong$30,000 live cells were used per condition. **b** Western blot analyses of hVA-MSC after 10 days of adipogenic induction with/out IFNγ (10 ng/ml) and/or STAT5i with the concentrations mentioned. **c** Confocal Z projection of *STAT5B* knocked down hVA-MSCs after 7 days of adipogenic stimulation with/out IFNγ (10 ng/ml) or TGFβ (10 ng/ml) with pSmad3 antibody. Far Red- Phalloidin (cyan) and DAPI (blue) were used for marking the cytoskeleton and nucleus, respectively. An image from one representative donor (*N* = 3) is shown. **d** Representative western blot analysis (*N* = 2) of hVA-MSC after 10 days of adipogenic induction with/out IFNγ (10 ng/ml) and/or Galunisertib (20 µM). **e** Representative western blot analysis of hVA-MSC cell lysates after 10 days of adipogenic induction with/out IFNγ (10 ng/ml). Proteasomal inhibitor MG132 was applied at 2 µM for the final 12 h. **f**, **g** Western blot quantification (*N* = 3) of hVA-MSCs after 10 days of adipogenic induction with/out IFNγ (10 ng/ml) and specific inhibitors as mentioned. **h**–**j** Co-immunoprecipitation analysis of hVA-MSC after 10 days of adipogenic induction with/out IFNγ (10 ng/ml) and/or Galunisertib (20 µM). Specific antibodies used for Co-IP are mentioned. **k** Schematic diagram representing STAT5- Smad3 interaction via Smad4 in VA-MSC under chronic inflammatory conditions. Ind-adipogenic induction. Normal cell culture media (Ind^−^IFN^−^) treated cells were used as the basal condition. For western blots, GAPDH was used as loading control. Scale- 100 µm. Error bars represent mean ± SEM. * indicates statistical significance (**P < 0.005; ***P < 0.0005; ****P < 0.00005) of Tukey’s test post one-way ANOVA.

Next, we sought to uncover the underlying molecular mechanism of IFNγ and TGFβ/Smad3 cooperativity. First, we tested if hVA-MSCs, like many other cells, themselves secrete TGFβ and whether this secretion is influenced by chronic IFNγ exposure. ELISA analysis of hVA-MSC cell culture media treated under various conditions show that hVA-MSCs indeed secrete TGFβ, but its concentration did not change by the presence of adipogenic cocktail, IFNγ, or small-molecule inhibitors (Supplementary Fig. 3a). Next, we tested whether IFNγ treatment alters cell-surface level expression of TGFBR1. Flow cytometry analysis of hVA-MSCs treated with adipogenic media for 10 days revealed that adipogenic stimulation decreased cell-surface level expression of TGFBR1 (Fig. 5a). IFNγ did not affect this TGFBR1 downregulation further, indicating that intracellular mechanism(s), rather than enhanced phosphorylation, is responsible for observed IFNγ prompted Smad3 activation.

As both STAT5 and Smad3 were found to be critical for VA-MSC differentiation inhibition, we next tested if STAT5 is responsible for the IFNγ-induced upregulation of pSmad3. Indeed, specific STAT5 inhibition by STAT5i decreased pSmad3 in a dose-dependent manner despite continuous presence of IFNγ (Fig. 5b). Like pharmacological inhibition, specific genetic knockdown of *STAT5B* caused downregulation of pSmad3 in IFNγ treated hVA-MSCs, further supporting the role of STAT5 in chronically augmenting pSmad3 (Supplementary Fig. 3b). Based on these data, we predicted that active STAT5 is necessary for pSmad3 signaling, and *STAT5* inhibition would diminish the transcriptional regulatory activity, as represented by the nuclear localization of phosphorylated pSmad3. Indeed, DSiRNA-mediated *STAT5B* knockdown resulted in reduced nuclear localization of pSmad3 in hVA-MSCs adipogenically stimulated in the presence of IFNγ or TGFβ (Fig. 5c).

We next tested whether TGFβ/Smad3 reciprocally modulates chronic STAT5 activation. For this, adipogenesis was induced in hVA-MSCs in the presence of IFNγ and/or TGFBR1 inhibitor Galunisertib for 10 days. Then, the cell lysates were subjected to western blot analysis using antibodies against active and total STAT proteins. Under these conditions, Galunisertib treatment only reduced pSTAT5 protein levels without affecting other STAT proteins, showing that TGFβ signaling is indeed required for chronic STAT5 activation (Fig. 5d).

Having identified crucial roles of STAT5–Smad3 synergy in maintaining high levels of these activated proteins, we next investigated the underlying molecular mechanism. Post phosphorylation-activation by cell-surface receptors, effector proteins undergo proteasomal degradation to naturally limit signal duration and strength. Conversely, inhibition of proteasomal degradation may allow sustained activation of signaling pathway(s) under chronic inflammatory stress. As STAT5 and Smad3 inhibition under chronic IFN or TGFβ treatment conditions caused reciprocal downregulation, we hypothesized that once concomitantly activated, these proteins protect each other from proteasomal degradation. To test this, hVA-MSCs were treated along with adipogenic cocktail for 10 days with/out IFNγ, and small-molecule proteasomal inhibitor MG132^45^ was applied for the last 12 h. Then we performed western blot with antibodies against TGFβ and IFNγ signaling pathway members (Fig. 5e). First, we observed that MG132 reversed adipogenic induction mediated pSmad3 downregulation and caused further accumulation of pSmad3 in IFNγ-treated cells. Second, MG132 caused increased accumulation of all phospho-STAT proteins under IFNγ treatment condition. However, unlike pSTAT1 or pSTAT3, pSTAT5 levels increased vastly.

Next, we confirmed these observations in 3–4 distinct donor-derived MSCs. For this, MSCs were treated with adipogenic stimulation conditions as above in the presence of IFNγ and/ or selective Smad3 inhibitor SIS3^46^, STAT5i, Galunisertib or MG132. As shown in Fig. 5f, pan TGFβ signaling inhibitor Galunisertib or Smad3 specific inhibitor SIS3 significantly downregulates IFNγ stimulated pSTAT5 levels, whereas proteasomal inhibitor MG132 significantly further upregulates IFNγ stimulated pSTAT5. On the other hand, MG132 application under adipogenic conditions significantly increased pSmad3, mimicking the action of IFNγ (Fig. 5g); this increase in IFNγ stimulated pSmad3 levels could be reversed significantly by inhibiting STAT5 via STAT5i. Taken together, these results indicate that IFNγ-activated pSTAT5 is degraded via proteasome and sustained STAT5 activation requires continuous IFNγ exposure, akin to a chronic inflammatory microenvironment. Chronically active STAT5, in turn, prevents pSmad3 proteasomal degradation required for adipogenic differentiation cascade to proceed.

Next, we investigated whether STAT5 physically interact with Smad3 or Smad4 to prevent pSmad3 degradation. For this, a series of co- immunoprecipitation assays were performed on hVA-MSCs chronically treated with IFNγ under adipogenic stimulation. After 10 days of treatment, cell lysates were collected under non-denaturing condition and co-IPs were performed using monoclonal antibodies against STAT5, Smad3, and Smad4 (Fig. 5h–j). We observed that Smad3 physically interacted with Smad4 but did not directly bind to STAT5 (Fig. 5h). On the other hand, STAT5 was found to physically interact with Smad4 but not directly with Smad3 (Fig. 5i). This STAT5–Smad4 interaction depended on Smad3-activation status as Galunisertib treatment diminished this interaction (Fig. 5i). No other STAT protein was found to interact with either STAT5 or Smad3. The observed Smad4–STAT5 and Smad4–Smad3 interaction was validated by a reciprocal co-IP against Smad4 (Fig. 5j).

Collectively, these results indicate that IFNγ activated pSTAT5 physically interacts with TGFβ activated pSmad3-Smad4 complex through Smad4; this interaction protects the complex from proteasomal degradation and allows to suppress adipogenic regeneration under chronic inflammatory conditions (Fig. 5k schematic).

### Human VA-MSC adipogenic inhibition by IFNγ and TGFβ is conserved in murine in vivo and in vitro models of chronic metabolic inflammation

**Fig. 6:**
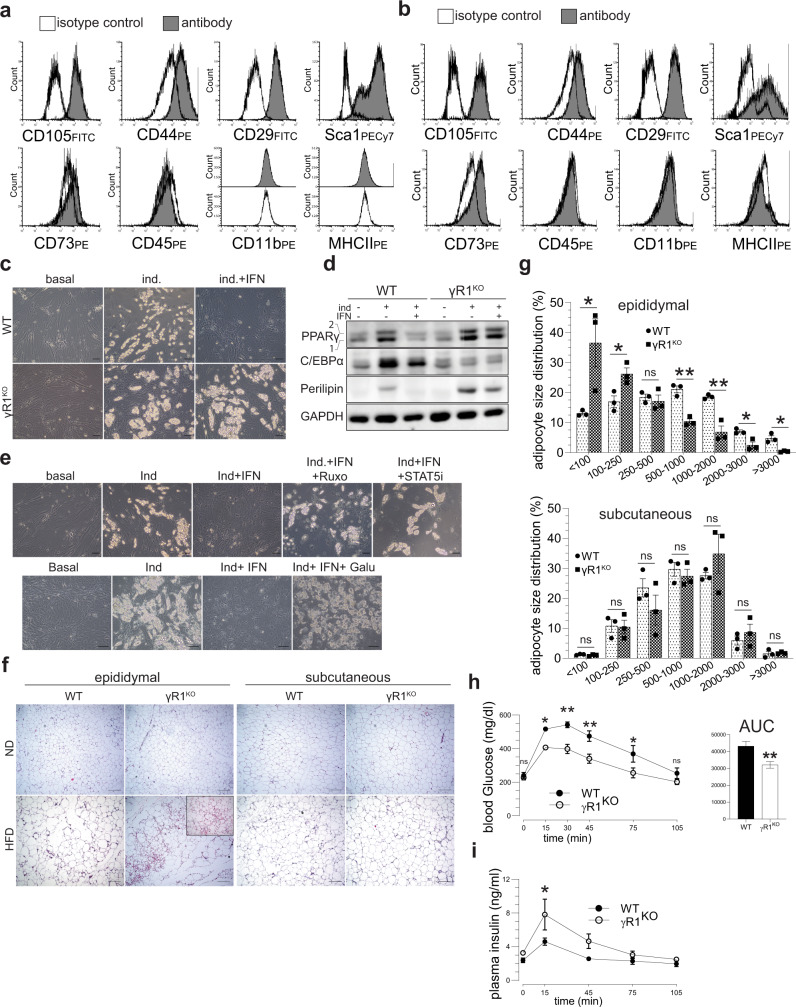
Murine model of VA-MSC adipogenic suppression under chronic inflammation supports the central role of STAT5–Smad3 synergy. **a**, **b** Representative (*N* = 3) flow cytometry Analysis of MSC-specific marker expression in WT mVA-MSC (**a**) and γR1KO-mVA-MSC (**b**). Unshaded peaks represent isotype control, shaded peaks represent cells stained with the antibodies mentioned. These cells show a marker phenotype of CD105^+^CD44^+^CD29^+^Sca1^+^CD73^−^CD45^−^CD11b^−^MHCII^−^. ~60,000–80,000 cells were used/sample. **c** Representative (*N* = 3) phase-contrast microscopy of mVA- MSCs after 7 days of adipogenic induction with/out murine IFNγ (10 ng/ml). Bright globules are lipid droplets. **d** Representative western blot analysis (*N* = 2) of mVA-MSC lysate after 7 days of adipogenic induction. Murine IFNγ was used at 10 ng/ml. **e** Representative (*N* = 3) phase-contrast microscopy of WT mVA-MSC after 7 days of adipogenic stimulation with/out murine IFNγ (10 ng/ml) with inhibitors (Ruxolitinib—5 µM; STAT5i—200 µM, Galunisertib—20 µM). **f**, **g** Representative H&E stained images (**f**) and quantification of adipocyte size (**g**, *N* = 3/group) from fat pads of age-matched (~25 weeks old), ND, and HFD-fed male animals. (Inset; γR1^KO^ HFD- higher magnification (×40) image of the hyperplasic region). **h** Oral glucose-tolerance test (oGTT) of ~25 weeks HFD-fed WT and γR1^KO^ animals. Area under curve (AUC) analysis of the curves are shown. *N* = 5 animals/group (**i**) plasma insulin measurement during oGTT of (**h**). Error bars represent mean ± SEM. * indicates statistical significance (*P < 0.05, **P < 0.005; ***P < 0.0005; ****P < 0.00005) of multiple unpaired *t* tests for (**g**); for (**h**–**i**), (*P < 0.05; **P < 0.005) of Sidak’s multiple comparison test followed by two-way ANOVA.

Having determined the molecular mechanism of hVA-MSC adipogenic regulation under inflammatory conditions, we next studied the evolutionarily conserved nature of our findings in a murine model system. For this, we used an IFNγ receptor 1 (IFNGR1) knockout mouse model (γR1^KO^)^47^ that lack the cytoplasmic domain of IFNGR1 and unable to transduce intracellular signal. Adipose tissue samples were collected from adult (~25-week-old male) “wild type” control (B6; WT) and γR1KO animal’s epididymal fat pads. Epididymal fat pads functionally resemble human visceral fat depots, therefore constitute a suitable murine model system to study adipogenesis. As before, the MSCs were isolated from stromal vascular fraction of epididymal fat pads, and specific identity of these cells were determined by flow cytometry (Fig. 6a, b). The cell-surface marker expression pattern, i.e., CD105^+^CD44^+^CD29^+^Sca1^+^CD73^−^CD45^−^CD11b^−^MHCII^−^, confirms the mouse MSC identity of these cells^19–22^.

Next, adipogenic differentiation in WT and γR1^KO^ mVA-MSCs were induced by incubating cells with an adipogenic cocktail and regular media change every 48 h. (Fig. 6c). Unlike human VA-MSCs, both types of mVA-MSCs showed prominent lipid droplet deposition only after 7 days of differentiation. As expected, concomitant IFNγ exposure (at 10 ng/ml) completely inhibited the formation of lipid droplets in WT MSC but not in γR1^KO^ MSCs. Next, we studied the molecular mechanism of adipogenic regulation in mVA-MSC. Western blot analysis revealed that chronic IFNγ exposure prevented PPARγ protein expression in WT mVA-MSCs (Fig. 6d). In contrast, γR1^KO^ MSCs were resistant to IFNγ and expressed PPARγ normally. However, we did not see any CEBPα upregulation in γR1^KO^ MSCs upon adipogenic stimulation but adipogenesis progressed normally, as evidenced by lipid droplet accumulation (Fig. 6c) and perilipin (a lipid-binding protein expressed in mature adipocytes) expression (Fig. 6d), suggesting that C/EBPα may not be necessary for adipogenesis, at least in mVA-MSCs. These data indicate that molecular mechanism of IFNγ mediated inhibition of adipogenic differentiation is evolutionarily conserved in mouse MSCs.

In order to determine whether, like hVA-MSC, STAT5 and Smad3 play integral role in preventing adipogenic differentiation in mVA-MSC under chronic inflammatory conditions, we induced differentiation in WT mVA-MSC with murine IFNγ and specific inhibitors (Fig. 6e). JAK2 inhibitor Ruxolitinib or STAT5 inhibitor STAT5i were able to restore adipogenesis, indicating that JAK2-STAT5 axis plays central role in IFNγ mediated differentiation inhibition. Similarly, IFNγ induced adipogenic inhibition could be reversed by concomitant Galunisertib treatment, showing that Smad3 comprise another evolutionarily conserved central node in mVA-MSC plasticity control under chronic inflammation.

To study the physiological relevance of our findings regarding chronic inflammatory conditions, we resorted to using a “western style” high-fat high-caloric (45 kcal% fat; referred as HFD). Freshly weaned γR1^KO^ and age-matched WT controls were maintained on ad libitum HFD diet for ~25 weeks. Next, we studied the degree of neoadipogenesis in various fat pads of HFD-fed animals and their age-matched, normal chow diet (ND) fed, counterparts. The predominant visceral WAT in rodents is epididymal fat^48^. Short-term metabolic stimulation leads to neoadipogenesis/hyperplasia in epididymal fat but chronic over-nutritional challenge prevents this process^49^. Histochemistry analysis of these fat pads revealed no appreciable differences between ND-fed WT and γR1^KO^ (Fig. 6f, epididymal-ND). However, upon long-term HFD feeding, WT epididymal fat pads showed only hypertrophic growth. In stark contrast, numerous smaller cells, representing newly differentiated adipocytes, could be observed alongside hypertrophic cells in γR1^KO^ fat pads (Fig. 6f, epididymal-HFD). Adipocyte size distribution analysis further confirmed this observation, where we observed significantly increased number of new adipocytes and significantly decreased the number of old adipocytes in γR1^KO^ fat pads (Fig. 6g). On the other hand, γR1^KO^ animals did not show enhanced adipogenesis or significant difference in adipocyte size profile compared to controls in the subcutaneous fat pads, which are known to be characteristically different than visceral fat depots (Fig. 6f, subcutaneous-HFD and g).

In order to determine whether IFNγ impeded neoadipogenesis affects systemic metabolism, we performed oral glucose-tolerance tests (oGTT) and measured insulin (the major circulating glucose regulating hormone released by islet beta cells in response to nutrient consumption) secretion during oGTT. For this, HFD-fed γR1^KO^ and WT mice were fasted for 6 h to achieve a baseline fasting glucose level. Then, they were given a body weight normalized oral bolus of glucose solution and blood samples were collected at regular intervals to measure circulating glucose and insulin concentrations. γR1^KO^ mice display improved glycemic control, as revealed by significantly reduced circulating glucose levels during oGTT, and significantly smaller “area under curve” compared to control (Fig. 6h). This comparatively lower circulating glucose levels during oGTT indicates efficient glucose adsorption by glucose-responsive tissues including adipose and muscle. Of note, although γR1^KO^ animals displayed somewhat increased secreted insulin levels during oGTT, differences were statistically insignificant for most of the time points tested (Fig. 6i). Flow cytometry analysis of stromal vascular fraction cells from the epididymal fat pads of HFD-fed mice showed that γR1^KO^ animals harbor significantly more MSC cells, as characterized by the MSC surface marker expression (Supplementary Fig. 4a, b), indicating that chronic inflammatory conditions may prevent VA-MSC self-renewal or induce apoptosis in an IFNγ dependent manner.

Collectively, these results show that the lack of functional IFNγ signaling confers protection from chronic HFD-induced metabolic dysfunction in γR1^KO^ animals, and sustained adipose regeneration may play a significant role in such protection.

**Fig. 7:**
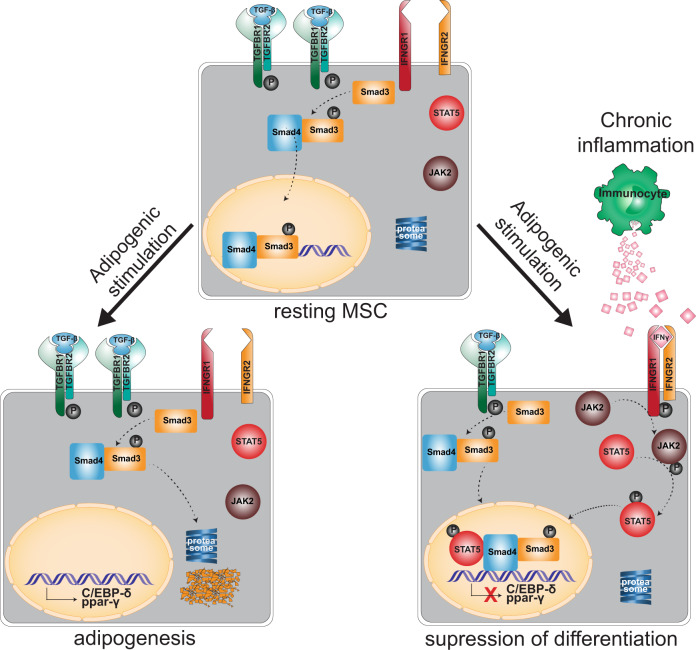
A model of chronic inflammation-induced inhibition of neoadipogenesis. Under normal adipogenic stimulatory conditions, phosphorylated, active Smad3 transcription factor is rapidly degraded via the proteasomal mechanism to allow the expression of key adipogenic δ transcription factors, including C/EBP-δ and PPAR-γ. In contrast, under chronic inflammatory conditions, IFNγ-activated STAT5 and TGFβ-activated Smad3 physically interact through Smad4. This complex is protected from proteasomal degradation and prevent adipogenic transcriptional cascade progression in VA-MSCs.

Based on these results, we propose a model of how chronic flammation affects VA-MSC adipogenic plasticity (Fig. 7). Under chronic inflammatory stress, IFNγ (secreted from infiltrating immunocytes) and TGFβ (secreted from adipocytes or MSCs themselves) synergistically act on VA-MSCs to supress adipogenic differentiation. Mechanistically, IFNγ activated STAT5 and TGFβ activated Smad3 transcription factors physically interact through Smad4. Consequently, this STAT5–Smad3 dyad prevents natural downregulation of Smad3 required for adipogenic cascade progression.

## Discussion

How chronic inflammatory microenvironment affects the neoadipogenic potential of VA-MSCs remain largely unexplored. We identified and characterized two key pro-inflammatory factors, namely IFNγ and TGFβ, that act in a coordinated manner to prevent human and mouse VA-MSC differentiation under such conditions.

IFNγ is a key mediator of inflammation in mouse and human adipose tissue^50–53^. However, the molecular mechanism of IFNγ’s inhibitory action in VA-MSC differentiation remains largely unknown. Here, we show that IFNγ inhibits early adipogenic commitment by selectively suppressing the expression of C/EBP-δ transcription factor which promotes the initial expression of PPARγ, the adipogenesis master regulator^29^ (Figs. 1 and 6).

So far, STAT1 transcription factor has been considered as the canonical IFNγ signal mediator in adipose tissue^54,55^. However, we show that long-term IFNγ exposure (5–14 days) resulted in the JAK2 mediated transcriptional upregulation as well as sustained activation and nuclear localization of STAT5, STAT3 and STAT1 proteins in VA-MSCs (Figs. 2, 3, and Supplementary Fig. 2). This upregulation of total STAT mRNA and protein levels are completely different than canonical IFNγ signaling, which causes rapid phosphorylation/activation followed by downregulation of active STATs without transcriptional upregulation. These observations underscore the fundamental differences between acute and chronic inflammatory functions of the IFNγ pathway, with the latter being more relevant in chronic inflammatory conditions. It is important to note that commonly used IFNγ concentrations for in vitro studies (in ng/ml range; for example, ref. ^55^) exceed that of in vivo acute or chronic inflammatory conditions (usually range in pg/ml). High-fat diet rodent model studies here provided validation of hVA-MSC results and assisted in understanding physiological roles of IFNγ signaling during adipogenesis.

Molecular characterization of IFNγ’s action identified STAT5, but not STAT1 or STAT3, as a critical regulator of adipogenic differentiation in both mVA and hVA-MSCs (Fig. 3 and Supplementary Fig. 2e, f). STAT5 is known to be activated by many cytokines, growth factors and interleukins^56^. However, to our knowledge, STAT5 has not been described as an effector of IFNγ so far. Several cytokines, shown to stimulate STAT5 in various immune cells, are also known to play inflammatory roles in adipose tissue, including TNFα, IL6, IL-1β, and IL2^57–64^. However, none of these factors were able to prevent hVA-MSC adipogenic differentiation (Supplementary Fig. 1), indicating that IFNγ specifically acts as a sensor of inflammatory conditions in VA-MSCs to regulate adipogenic plasticity through STAT5.

Our STAT5-related observations do not directly match with some previous reports^65,66^ where STAT5 positively regulated adipogenesis. There could be several reasons for this discrepancy. First, adult human and mouse multipotent visceral MSCs used in our work constitute vastly different model systems than NIH3T3 and 3T3-L1 cell lines which were originated from mouse embryonic fibroblasts. Second, we mainly focused on the natural augmentation of STAT5 by IFNγ and did not employ STAT5 overexpression. It is indeed possible that overexpression-mediated upregulation of unphosphorylated STAT5 is beneficial for adipogenesis. Further studies will be needed to answer these questions.

In addition to IFNγ, our screen identified TGFβ as an inhibitor of adipogenesis (Fig. 4a and Supplementary Fig 1). Several previous reports identified TGFβ as an inhibitor of adipogenesis in preadipocytes and in vivo^43,67,68^. However, the natural regulation of this pathway during adipogenesis, and its chronic effects on VA-MSc have not been investigated. We show that TGFβ signaling is active in resting VA-MSCs. Adipogenic stimulation reduces cell-surface expression of TGFBR1, resulting in a downregulation of Smad3 branch of this pathway (Fig. 4). Furthermore, we found that IFNγ acts through STAT5 to prevent this Smad3 downregulation upon chronic inflammation (Fig. 5), where active STA5 and Smad3 physically interact via Smad4 to protect the complex from proteasomal degradation. Although some direct interaction between STAT and Smad proteins, such as STAT3 and Smad1, has been reported before^69^, to our knowledge STAT5–Smad4-Smad3 interaction was unknown. These results collectively indicate that STAT5–Smad3 dyad comprises a highly specific node of chronic inflammation in VA-MSCs where multiple signaling pathways act in concert to exert sustained anti- adipogenic functions. Pharmacological inhibition of Smad3 or STAT5 under such chronic inflammatory conditions allowed normal adipose regeneration in both mVA and hVA-MSCs. This may provide a potential therapeutic strategy for restoring neoadipogenesis in patients with insulin resistance or metabolic syndrome.

Loss of adipose tissue functionality is linked with various “wasting syndromes”^12–14,70^. These syndromes, such as cachexia and sarcopenia, manifest upon long-term systemic inflammatory responses that accompany chronic diseases, including cancer, chronic kidney disease, thyroid disease, chronic liver failure, etc. In addition to maintaining energy homeostasis, healthy adipocytes secrete adipokines, such as leptin, that co-ordinate metabolism, muscle growth, and myocardial health^70,71^. Our results indicate that pharmacological restoration of adipogenesis is possible under chronic inflammatory conditions by either inhibiting pan JAK-STAT and TGFβ pathways or specifically impairing STAT5–Smad3 synergy. These observations may provide therapeutic guidance to improve systemic metabolism in patients with wasting syndromes.

Finally, STAT5–Smad3 synergy may have implications beyond adipogenesis and metabolic dysfunction. For example, aberrant activation (but not mutation) of STAT5 has been linked with cell survival, tumorigenesis, and malignancy in a number of primary human tumors^56^. Similarly, specific upregulation of Smad3 has been linked with aggressive triple-negative breast cancer^72^. It is possible that STAT5–Smad3 dyad play critical role(s) in these cells by allowing prolonged, enhanced activity of these transcription factors that leads to tumorigenesis and malignancy. Further studies will be needed to shed light on these aspects of IFNγ- TGFβ signaling co-operativity.

## Methods

### Study approval

All animal experiments were approved by the University of Wisconsin-Madison Institutional Animal Care and Use Committee and performed in accordance with the Animal Care and Use Policies of the University of Wisconsin-Madison (IACUC ID - M006496).

All human tissue collections were done from deidentified, consenting individuals upon University of Wisconsin-Madison Institutional Review Board approval (IRB ID- 2016-1545).

### Mouse husbandry

*C57BL/6J* (B6, stock no.000664) and *B6.129S7-Ifngr1tm1Agt/J* (γR1KO, stock no. 003288) were obtained from the Jackson Laboratory (Bar Harbor, ME). Animals were bred and maintained in a level 2 specific pathogen-free facility with 12 h light/dark cycle and ad libitum access to standard mouse chow diet and water.

### HFD feeding and in vivo experiments

After weaning, 4-weeks-old male animals were placed on “western style” high-fat, high-carbohydrate Envigo Teklad diet (catalog no. TD.06415). Mice were fed this diet for ~25 weeks ad libitum. Random, non-fasting body weight and blood glucose were measured at a regular, weekly interval.

Oral glucose-tolerance test (oGTT)—animals were fasted for 6 h with free access to water. Then blood glucose concentration was measured and animals were given 2 gm glucose/kg body weight by oral gavage using freshly prepared 20% glucose solution in saline. Circulating glucose concentrations were measured at regular intervals. Throughout the experiment, animals had free access to water.

Plasma insulin concertation measurement during oGTT—~50 µl blood was collected at regular intervals during oGTT using heparinized capillary tube. Plasma was isolated, and insulin concentration was determined using Crystal Chem Ultra-Sensitive Mouse Insulin ELISA Kit according to the manufacturer’s instructions.

Plasma IFNγ measurement—~100 µl tail vein blood was collected from HFD-fed animals using a heparinized capillary tube. Plasma was isolated, and IF-γ concentration was determined using Thermo-Fischer IFNγ sandwich ELISA kit according to the manufacturer’s instructions.

### Adipocyte size measurement

H&E stained WT, and YR1^KO^ fat pad sections (epididymal and subcutaneous, taken under identical magnification) were used for adipocyte size measurement using Adiposoft plugin^73^ of ImageJ software.

### Mouse epididymal adipose MSC isolation

MSC were isolated from stromal vascular fraction as described in ref. ^74^ with modifications. In short, epididymal fat pads from ~20-weeks-old animals (3–4 animals/group) were collected under sterile condition and cut into small pieces (~1 mm^3^) in HBSS. Tissues were further digested using 2 mg/ml collagenase type IV for 30 min in 37 °c water bath with intermittent shaking until the solution appears homogeneous by visual inspection. Cells were washed twice in HBSS and filtered through a 100-µm mesh filter. This stromal vascular fraction was plated on 150-cm^2^ plate in α-MEM medium supplemented with 20% FBS + 12.5 mM l-glutamine+ 1% penicillin–streptomycin. Media was changed with fresh media after 48 h to remove all non-adherent cells. Cells were passaged in 1:3 ratio after reaching ~90% confluency. MSC identity of these cells was measured during passage 2 by flow cytometry. For experimental purposes, only passage 2–6 cells were used.

### Human intra-abdominal adipose MSC isolation

Intra-abdominal adipose tissue sections (~3 × 3 × 2 cm) were collected from deidentified healthy consenting donors. MSCs were isolated as described in ref. ^75^. Media was changed with fresh media after 48 h to remove all non-adherent cells. Upon reaching 70% confluence, cells were split in 1:5 ratio and cultured in DMEM + 10% human platelet lysate (HPL) + l-glutamine+ penicillin–streptomycin. After two passages, MSC identity of these cells was measured by flow cytometry. For experimental purposes, only passage 2–6 cells were used for all assays.

### Adipogenic induction of mouse visceral adipose MSC

Cells were seeded at 5000/cm^2^ density and allowed to become ~70% confluent in α-MEM–20%FBS media. Media was then changed to adipogenic basal media (α-MEM/10% FBS/L-glutamine/pen-strep). Then, the media was changed with adipogenic induction cocktail (day 0) prepared in basal media according to Supplementary Table 1. These concentrations were determined based on^76^ and preliminary experiments performed to determine the most robust adipogenesis-producing condition. Media was changed every 48 h until day 7. DMSO was used as vehicle control for the basal condition.

### Adipogenic induction of human visceral adipose MSC

Cells were seeded at 5000/cm^2^ density and allowed to become ~70% confluent in DMEM-10%HPL/L-glutamine/pen-strep media and then changed to adipogenic basal media (DMEM/10% FBS/L-glutamine/pen-strep). After 24 h, media was changed with adipogenic induction cocktail (day 0) prepared according to supplementary Table 2. These concentrations were determined based on ref. ^76^ and preliminary experiments performed to determine the most robust adipogenesis-producing condition. Media was changed every 48/72 h until day 14.

### hVA-MSC adipogenesis screen

hVA-MSCs were seeded at 5000/cm^2^ density in 12-well plates. Adipogenesis was induced as above. The cells were concomitantly treated with individual cytokine/chemokine/growth factors as shown in Supplementary Fig 1. The range of concentrations of these factors, i.e., 1 ng/ml–30 ng/ml, was chosen based on literature study to encompass a range of commonly used concentrations of specific factors in immunocytes or preadipocyte cell lines (for example, please see references mentioned in ref. ^16^). For each factor, stock solutions were prepared according to the manufacturer recommendation, and aliquots were stored at −80 °C. Each aliquot was used only once. For dilution of all factors, culture medium was used. Media, along with specific factors, were replenished every 48 h. After 14 days of incubation, Oil Red staining was performed to determine adipogenesis, and micrographs were taken at the center of each well.

### Cytokine/interleukin treatment

Species compatibility was strictly maintained throughout all assays. Recombinant human or mouse cytokines/growth factors/interleukins were used for hVA or mVA-MSC experiments, respectively. Unless otherwise specified, IFNγ was used at 10 ng/ml (~0.5 nM) concentration throughout. All factors, along with basal or adipogenic induction medium were changed every 48 h.

### Small-molecule inhibitor/reagent treatment

All inhibitors were prepared according to the manufacturer’s recommendations. Specific inhibitors were freshly included in basal media or adipogenic induction cocktail every 48 h as applicable.

### Western blot

Cells were washed once with ice-cold PBS and lysed on plate on ice using Cell Signaling technology lysis buffer supplemented with 1 mM PMSF right before use. Lysates were sonicated on ice, centrifuged at 12,000×*g* for 15 min at 4 °c, and the supernatants were collected. Pierce™ 660 nm Protein Assay Reagent was used to determine protein concertation. Western blots were performed according to standard protocol. Primary antibodies were prepared in TBST + 5% BSA or 5%blotto according to the manufacturer’s recommendation. Images were taken using Amersham ImageQuant 800 imgaing system. Supplementary Table 4 lists all antibodies used for western blotting. All blots in a given image is derived from the same experiment, and they were processed in parallel. We have included original western blot chemiluminescent images with corresponding light micrographs showing molecular weight markers for all western blots in the supplementary document.

### DSiRNA knockdown

Duplex oligonucleotides targeting human *STAT5B, STAT1,* and *SMAD3* were obtained from Integrated DNA Technologies. DsiRNA universal negative control or DSi targets were transfected in VA-MSCs using lipofectamine3000 (Invitrogen) per manufacturer recommendation. Specific treatments were performed 24 h post-transfection with fresh media change. Supplementary Table 6 lists all DSiRNAs used in this study.

### qRT-PCR

The total RNA was isolated from MSCs using Qiagen RNeasy Mini Kit according to manufacturer protocol. cDNA was prepared from 1 µg total mRNA using Qiagen QuantiTect Reverse Transcription Kit according to the manufacturer’s protocol. cDNA samples were used for Real-time PCR using Qiagen QuantiTect SYBR^®^ Green PCR Kit according to manufacturer protocol. Primer sets were designed using IDT Primer Quest program. Delta delta Ct method was used for data presentation. *GAPDH* was used as housekeeping gene. Bio-Rad CFX Connect Real-Time PCR Detection System was used for qRT-PCR. supplementary Table 3 shows the primer sequences used.

### Flow cytometry

Cells were detached using accutase treatment and washed once with PBS. Cells were then washed twice in FACS buffer (PBS + 5%FBS + 0.09% sodium azide) and incubated with primary antibodies for 30 min at 4 °C. Cells were then washed twice again in FACS buffer and analyzed using Attune Nxt flow cytometer. Cell viability staining was done with DAPI or GhostRed780 dye according to standard method. Supplementary Table 4 lists all antibodies used for flow cytometry.

### Immunofluorescence/confocal imaging

MSCs were plated at 2000/cm^2^ density on sterile, non-TC coated, 1.5H precision coverslips and treated according to specific experimental requirements. Cells were fixed with 4% formaldehyde for 10 min at RT, Immunostaining was done according to the antibody manufacturer’s protocol. DNA was counterstained with DAPI. Coverslips were mounted using ProLong Diamond antifade mountant. Confocal images were taken using a Nikon A1rs HD Confocal Microscope at ×20 magnification. Identical instruments and acquisition settings were used for each set of experiments. Images were processed using Nikon Elements software. Supplementary Table 4 lists all antibodies used for immunofluorescence.

### Oil red staining

Cells were plated at 5000/cm^2^ density on a six-well plate, and adipogenesis was induced as described. Then cells were fixed for overnight at 4 °C in PBS + 4% formaldehyde. This overnight fixing prevents dispersal of lipid droplets during subsequent wash steps. Wells were then washed slowly but thoroughly in distilled water, incubated with 60% isopropanol (in water) for 2 min and then incubated with freshly prepared and filtered 60% Oil Red solution (isopropanol stock solution diluted in water) for 15 min at room temperature with slow rocking. Then wells were washed thoroughly in distilled water to remove excess Oil Red. Finally, cells were treated with hematoxylin solution to stain nuclei (as applicable). Microscopy images were taken under a phase-contrast setting at ×10–20 magnification using Zeiss Vert1A microscope equipped with Axiocam 305 color camera. Zen3.1 software was used for image processing.

### Triglyceride assay

Cells were plated at 5000/cm^2^ density, and adipogenesis was induced as described. Then cells were lysed using PBS + 1% triton X100. Triglyceride concentration was determined using Thermo Scientific™ Triglycerides Reagent according to the manufacturer’s protocol. A C2-C10 triglyceride mix was used to generate the standard curve. Total protein concentration was measured using Pierce 660 nm protein assay.

### ELISA

All ELISA were done according to the manufacturer’s protocol. Supplementary Table 5 list all ELISA kits used in this study.

### CO-IP

Cells were detached using accutase treatment and lysed using CST 1X lysis buffer on ice. Samples were sonicated briefly on ice and centrifuged at 12,000×*g* for 15 min at 4 °C, and the supernatants were collected. Protein concentration was measured using Pierce 660 nm protein assay. ~300 µg protein was used per IP experiment. Lysates were pre-cleared at 4 °C for 1 h. with Pierce™ Protein A/G Magnetic Beads and then incubated with primary antibodies according to the manufacturer’s guideline at 4 °C overnight with end-to-end rotation. Pierce™ Protein A/G Magnetic Beads were applied at 30 µl/IP tube and further incubated for 3 h at 4 °C. Beads were washed three times in TBST + 0.1% TritonX100 supplemented with protease and phosphatase inhibitors. Finally, proteins were eluated by adding 50 µl 1× Lameli buffer and incubating beads at 95 °C for 10 min with intermittent mild agitation. Elutions were run on 10% gel, and western blot was done as described before. Supplementary Table 4 lists all antibodies used for CO-IP.

### Reporting summary

Further information on research design is available in the Nature Research Reporting Summary linked to this article.

## Supplementary information

supplementary material

REPORTING SUMMARY

## Data Availability

Data files are available from the corresponding author on reasonable request.

## References

[1] Vegiopoulos A, Rohm M, Herzig S (2017). Adipose tissue: between the extremes. EMBO J..

[2] Ouchi N, Parker JL, Lugus JJ, Walsh K (2011). Adipokines in inflammation and metabolic disease. Nat. Rev. Immunol..

[3] Muir LA (2016). Adipose tissue fibrosis, hypertrophy, and hyperplasia: correlations with diabetes in human obesity. Obes..

[4] Verboven K (2018). Abdominal subcutaneous and visceral adipocyte size, lipolysis and inflammation relate to insulin resistance in male obese humans. Sci. Rep..

[5] Despres JP (2006). Is visceral obesity the cause of the metabolic syndrome?. Ann. Med..

[6] Salans LB, Cushman SW, Weismann RE (1973). Studies of human adipose tissue. Adipose cell size and number in nonobese and obese patients. J. Clin. Investig..

[7] Spalding KL (2008). Dynamics of fat cell turnover in humans. Nature.

[8] Tang W (2008). White fat progenitor cells reside in the adipose vasculature. Science.

[9] Jeffery E, Church CD, Holtrup B, Colman L, Rodeheffer MS (2015). Rapid depot-specific activation of adipocyte precursor cells at the onset of obesity. Nat. Cell Biol..

[10] Kim SM (2014). Loss of white adipose hyperplastic potential is associated with enhanced susceptibility to insulin resistance. Cell Metab..

[11] Grunberg JR (2017). Overexpressing the novel autocrine/endocrine adipokine WISP2 induces hyperplasia of the heart, white and brown adipose tissues and prevents insulin resistance. Sci. Rep..

[12] Lena A (2021). Sarcopenia and cachexia in chronic diseases: from mechanisms to treatment. Pol. Arch. Intern. Med..

[13] Mannelli, M., Gamberi, T., Magherini, F. & Fiaschi, T. The adipokines in cancer cachexia. *Int. J. Mol. Sci.* **21**, 4860 (2020). 10.3390/ijms21144860 PMC7402301 32660156

[14] Dalal S (2019). Lipid metabolism in cancer cachexia. Ann. Palliat. Med..

[15] Ruiz-Ojeda, F. J., Ruperez, A. I., Gomez-Llorente, C., Gil, A. & Aguilera, C. M. Cell models and their application for studying adipogenic differentiation in relation to obesity: a review. *Int. J. Mol. Sci.* **17**, 1040 (2016). 10.3390/ijms17071040 PMC4964416 27376273

[16] Jiang N, Li Y, Shu T, Wang J (2019). Cytokines and inflammation in adipogenesis: an updated review. Front. Med..

[17] Cawthorn WP, Scheller EL, MacDougald OA (2012). Adipose tissue stem cells meet preadipocyte commitment: going back to the future. J. Lipid Res..

[18] Weiss ARR, Dahlke MH (2019). Immunomodulation by mesenchymal stem cells (MSCs): mechanisms of action of living, apoptotic, and dead MSCs. Front. Immunol..

[19] Dominici M (2006). Minimal criteria for defining multipotent mesenchymal stromal cells. The International Society for Cellular Therapy position statement. Cytotherapy.

[20] Viswanathan S (2019). Mesenchymal stem versus stromal cells: International Society for Cell & Gene Therapy (ISCT(R)) Mesenchymal Stromal Cell committee position statement on nomenclature. Cytotherapy.

[21] Moll G (2019). Intravascular mesenchymal stromal/stem cell therapy product diversification: time for new clinical guidelines. Trends Mol. Med..

[22] Moll G, Ankrum JA, Olson SD, Nolta JA (2022). Improved MSC minimal criteria to maximize patient safety: a call to embrace tissue factor and hemocompatibility assessment of MSC products. Stem Cells Transl. Med..

[23] Cawthorn WP, Heyd F, Hegyi K, Sethi JK (2007). Tumour necrosis factor-alpha inhibits adipogenesis via a beta-catenin/TCF4(TCF7L2)-dependent pathway. Cell Death Differ..

[24] Gagnon A, Foster C, Landry A, Sorisky A (2013). The role of interleukin 1beta in the anti-adipogenic action of macrophages on human preadipocytes. J. Endocrinol..

[25] Almuraikhy S (2016). Interleukin-6 induces impairment in human subcutaneous adipogenesis in obesity-associated insulin resistance. Diabetologia.

[26] Almendro V (2009). Interleukin-15 increases calcineurin expression in 3T3-L1 cells: possible involvement on in vivo adipocyte differentiation. Int. J. Mol. Med..

[27] O’Shea JJ (2015). The JAK-STAT pathway: impact on human disease and therapeutic intervention. Annu. Rev. Med..

[28] Ivashkiv LB (2018). IFNgamma: signalling, epigenetics and roles in immunity, metabolism, disease and cancer immunotherapy. Nat. Rev. Immunol..

[29] Hishida T, Nishizuka M, Osada S, Imagawa M (2009). The role of C/EBPdelta in the early stages of adipogenesis. Biochimie.

[30] Brun RP (1996). Differential activation of adipogenesis by multiple PPAR isoforms. Genes Dev..

[31] Yeh WC, Cao Z, Classon M, McKnight SL (1995). Cascade regulation of terminal adipocyte differentiation by three members of the C/EBP family of leucine zipper proteins. Genes Dev..

[32] Morris R, Kershaw NJ, Babon JJ (2018). The molecular details of cytokine signaling via the JAK/STAT pathway. Protein Sci..

[33] Bhat MY (2018). Comprehensive network map of interferon gamma signaling. J. Cell Commun. Signal.

[34] Castro F, Cardoso AP, Goncalves RM, Serre K, Oliveira MJ (2018). Interferon-gamma at the crossroads of tumor immune surveillance or evasion. Front. Immunol..

[35] Quintas-Cardama A (2010). Preclinical characterization of the selective JAK1/2 inhibitor INCB018424: therapeutic implications for the treatment of myeloproliferative neoplasms. Blood.

[36] Muller J, Sperl B, Reindl W, Kiessling A, Berg T (2008). Discovery of chromone-based inhibitors of the transcription factor STAT5. Chembiochem.

[37] Delgoffe GM, Vignali DA (2013). STAT heterodimers in immunity: a mixed message or a unique signal?. JAKSTAT.

[38] Torella D (2007). Fludarabine prevents smooth muscle proliferation in vitro and neointimal hyperplasia in vivo through specific inhibition of STAT-1 activation. Am. J. Physiol. Heart Circ. Physiol..

[39] Schust J, Sperl B, Hollis A, Mayer TU, Berg T (2006). Stattic: a small-molecule inhibitor of STAT3 activation and dimerization. Chem. Biol..

[40] Samad F, Yamamoto K, Pandey M, Loskutoff DJ (1997). Elevated expression of transforming growth factor-beta in adipose tissue from obese mice. Mol. Med.

[41] Alessi MC (2000). Plasminogen activator inhibitor 1, transforming growth factor-beta1, and BMI are closely associated in human adipose tissue during morbid obesity. Diabetes.

[42] Hata, A. & Chen, Y. G. TGF-beta signaling from receptors to smads. *Cold Spring Harb. Perspect. Biol.* **8**, a022061 (2016). 10.1101/cshperspect.a022061 PMC5008074 27449815

[43] Tsurutani Y (2011). The roles of transforming growth factor-beta and Smad3 signaling in adipocyte differentiation and obesity. Biochem Biophys. Res. Commun..

[44] Holmgaard RB (2018). Targeting the TGFbeta pathway with galunisertib, a TGFbetaRI small molecule inhibitor, promotes anti-tumor immunity leading to durable, complete responses, as monotherapy and in combination with checkpoint blockade. J. Immunother. Cancer.

[45] Palombella VJ, Rando OJ, Goldberg AL, Maniatis T (1994). The ubiquitin-proteasome pathway is required for processing the NF-kappa B1 precursor protein and the activation of NF-kappa B. Cell.

[46] Jinnin M, Ihn H, Tamaki K (2006). Characterization of SIS3, a novel specific inhibitor of Smad3, and its effect on transforming growth factor-beta1-induced extracellular matrix expression. Mol. Pharm..

[47] Huang S (1993). Immune response in mice that lack the interferon-gamma receptor. Science.

[48] Chusyd DE, Wang D, Huffman DM, Nagy TR (2016). Relationships between rodent white adipose fat pads and human white adipose fat depots. Front. Nutr..

[49] Jeffery E (2016). The adipose tissue microenvironment regulates depot-specific adipogenesis in obesity. Cell Metab..

[50] Rocha VZ (2008). Interferon-gamma, a Th1 cytokine, regulates fat inflammation: a role for adaptive immunity in obesity. Circ. Res..

[51] O’Rourke RW (2012). Systemic inflammation and insulin sensitivity in obese IFN-gamma knockout mice. Metabolism.

[52] Reardon CA (2018). Obesity and insulin resistance promote atherosclerosis through an IFNgamma-regulated macrophage protein network. Cell Rep..

[53] O’Rourke RW (2009). Depot-specific differences in inflammatory mediators and a role for NK cells and IFN-gamma in inflammation in human adipose tissue. Int. J. Obes..

[54] Cox AR (2020). STAT1 dissociates adipose tissue inflammation from insulin sensitivity in obesity. Diabetes.

[55] McGillicuddy FC (2009). Interferon gamma attenuates insulin signaling, lipid storage, and differentiation in human adipocytes via activation of the JAK/STAT pathway. J. Biol. Chem..

[56] Rani A, Murphy JJ (2016). STAT5 in cancer and immunity. J. Interferon Cytokine Res..

[57] Makki K, Froguel P, Wolowczuk I (2013). Adipose tissue in obesity-related inflammation and insulin resistance: cells, cytokines, and chemokines. ISRN Inflamm..

[58] Hotamisligil GS (2017). Inflammation, metaflammation and immunometabolic disorders. Nature.

[59] Jiang Y (2019). TNF-alpha enhances Th9 cell differentiation and antitumor immunity via TNFR2-dependent pathways. J. Immunother. Cancer.

[60] Han MS (2020). Regulation of adipose tissue inflammation by interleukin 6. Proc. Natl Acad. Sci. USA.

[61] Tormo AJ (2012). IL-6 activates STAT5 in T cells. Cytokine.

[62] Kochumon S (2020). Elevated adipose tissue associated IL-2 expression in obesity correlates with metabolic inflammation and insulin resistance. Sci. Rep..

[63] Bauche, D. et al. IL-23 and IL-2 activation of STAT5 is required for optimal IL-22 production in ILC3s during colitis. *Sci. Immunol.* **5**, eaav1080 (2020). 10.1126/sciimmunol.aav1080 32332067

[64] Gilmour KC, Pine R, Reich NC (1995). Interleukin 2 activates STAT5 transcription factor (mammary gland factor) and specific gene expression in T lymphocytes. Proc. Natl Acad. Sci. USA.

[65] Nanbu-Wakao R (2002). Stimulation of 3T3-L1 adipogenesis by signal transducer and activator of transcription 5. Mol. Endocrinol..

[66] Floyd ZE, Stephens JM (2003). STAT5A promotes adipogenesis in nonprecursor cells and associates with the glucocorticoid receptor during adipocyte differentiation. Diabetes.

[67] Ignotz RA, Massague J (1985). Type β transforming growth factor controls the adipogenic differentiation of 3T3 fibroblasts. Proc. Natl Acad. Sci. USA.

[68] Turner NJ, Jones HS, Davies JE, Canfield AE (2008). Cyclic stretch-induced TGFbeta1/Smad signaling inhibits adipogenesis in umbilical cord progenitor cells. Biochem Biophys. Res. Commun..

[69] Luo, K. Signaling cross talk between TGF-beta/Smad and other signaling pathways. *Cold Spring Harb. Perspect. Biol.* **9**, a022137 (2017). 10.1101/cshperspect.a022137 PMC5204325 27836834

[70] Yang, Q., Yan, C., Wang, X. & Gong, Z. Leptin induces muscle wasting in a zebrafish kras-driven hepatocellular carcinoma (HCC) model. *Dis. Model Mech.* **12**, dmm038240 (2019). 10.1242/dmm.038240 PMC6398506 30718259

[71] Parimisetty A (2016). Secret talk between adipose tissue and central nervous system via secreted factors-an emerging frontier in the neurodegenerative research. J. Neuroinflam..

[72] Singha PK (2019). Increased Smad3 and reduced Smad2 levels mediate the functional switch of TGF-beta from growth suppressor to growth and metastasis promoter through TMEPAI/PMEPA1 in triple negative breast cancer. Genes Cancer.

[73] Galarraga M (2012). Adiposoft: automated software for the analysis of white adipose tissue cellularity in histological sections. J. Lipid Res..

[74] Kilroy G, Dietrich M, Wu X, Gimble JM, Floyd ZE (2018). Isolation of murine adipose-derived stromal/stem cells for adipogenic differentiation or flow cytometry-based analysis. Methods Mol. Biol..

[75] Chinnadurai R (2019). Potency analysis of mesenchymal stromal cells using a phospho-STAT matrix loop analytical approach. Stem Cells.

[76] Scott MA, Nguyen VT, Levi B, James AW (2011). Current methods of adipogenic differentiation of mesenchymal stem cells. Stem Cells Dev..

