# Supplementary material for: A STAT5-Smad3 dyad regulates adipogenic plasticity of visceral adipose mesenchymal stromal cells during chronic inflammation
Source: NPJ Regen Med. 2022 Aug 31;7:41. doi: 10.1038/s41536-022-00244-5 (PMC9433418; doi:10.1038/s41536-022-00244-5)
Supplement: Supplementary file 1 — supplementary material [file 41536_2022_244_MOESM1_ESM.pdf]

Supplementary figures and tables

This section contains five supplementary figures (1-5), full blots and corresponding molecular weight marker images of the blot strips displayed in figures, and 6 supplementary tables detailing the reagents used in this study.

Supplementary Figures

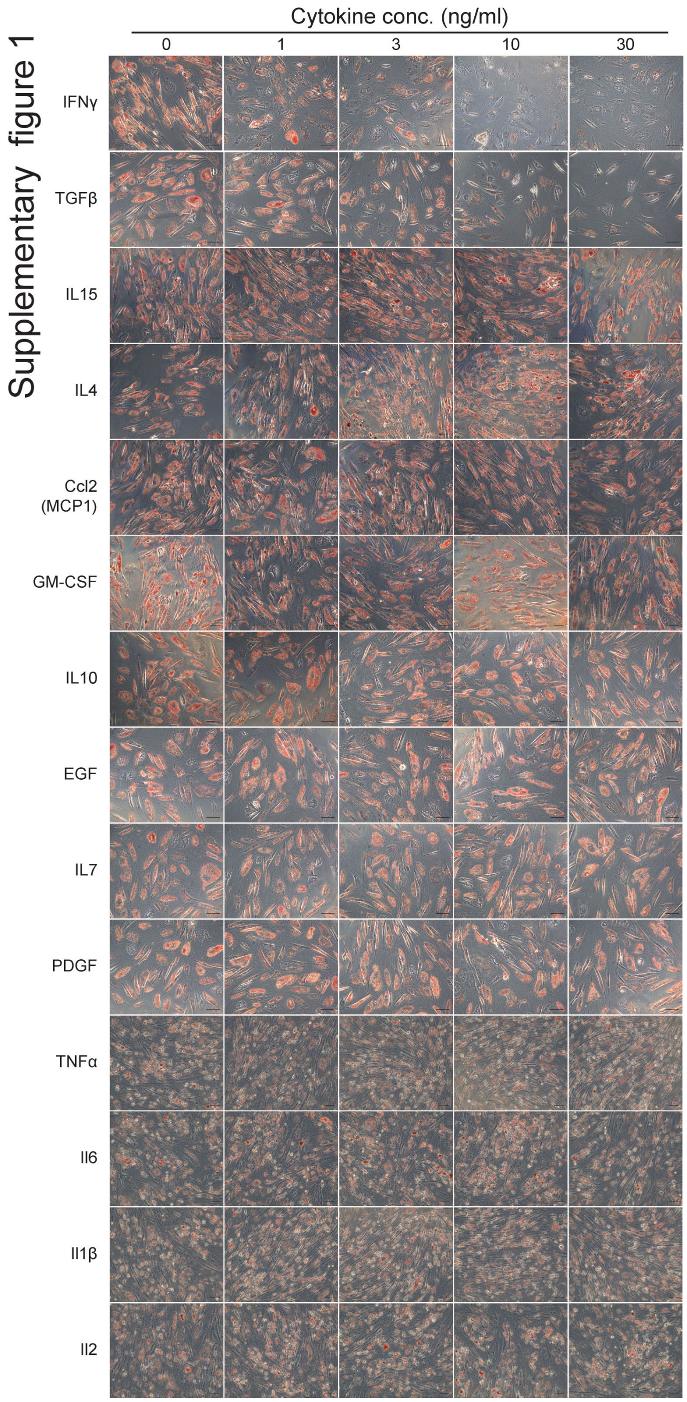

Supplementary Figure 1. hVA-MSC adipogenesis screen; Representative (N=3) Phase contrast microscopy of Oil red stained hVA-MSCs after 14 days of adipogenic induction with/out recombinant human cytokines as mentioned. Media, along with cytokines were changed every 48 hrs. Scale bar- 100μm.

Supplemtary figure 2

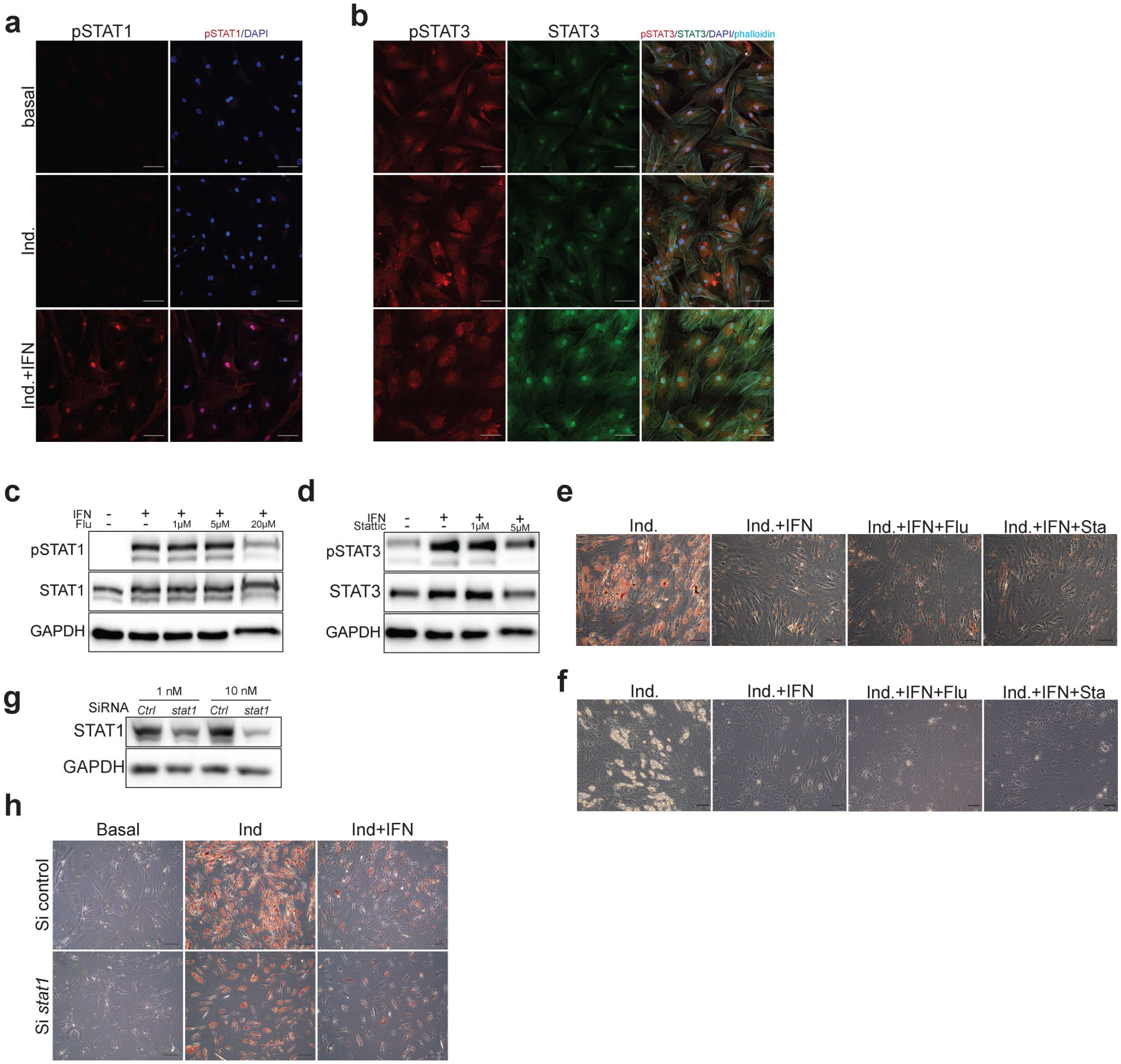

Supplementary Figure 2. STAT1 or STAT3 does not regulate IFNγ induced adipogenic inhibition in VA-MSCs

(a-b) Confocal Z projection of hVA-MSC stained after 10 days of adipogenic induction with/out human IFNγ (10ng/ml) with the specific antibodies mentioned. Phalloidin and DAPI was used for marking cytoskeleton and nucleus, respectively. Image from one representative donor (N=3) is shown. (c-d) Western blot analysis of hVA-MSCs after 10 days of adipogenic induction with/out IFNγ (10ng/ml) with the concentration of inhibitors mentioned. Flu-fludarabine, Sta-Stattic. (e) Representative (N=3) phase contrast microscopy of Oil red stained hVA-MSCs after 14 days of adipogenic induction with/out IFNγ (10ng/ml) with specific inhibitors mentioned (Fludarabine- 20 µM, Stattic-5µM). (f) Representative (N=3) Phase contrast microscopy of WT mVA-MSC after 7 days of adipogenic stimulation with/out murine IFNγ (10ng/ml) with inhibitor concentrations as above. (g) Western blot analysis of *STAT1* knocked down hVA-MSCs after 7 days of treatment with IFNγ (10ng/ml). Concentration of the DsiRNAs are mentioned. (h) Representative (N=3)

phase contrast microscopy of Oil red stained hVA-MSCs after *STAT1* knock down (at 10nM DSiRNA concentration) and 14 days of adipogenic induction with/out human IFNγ (10ng/ml). Ind-adipogenic induction. For western blots, GAPDH was used as loading control. Scale- 100μm.

Supplemtary figure 3

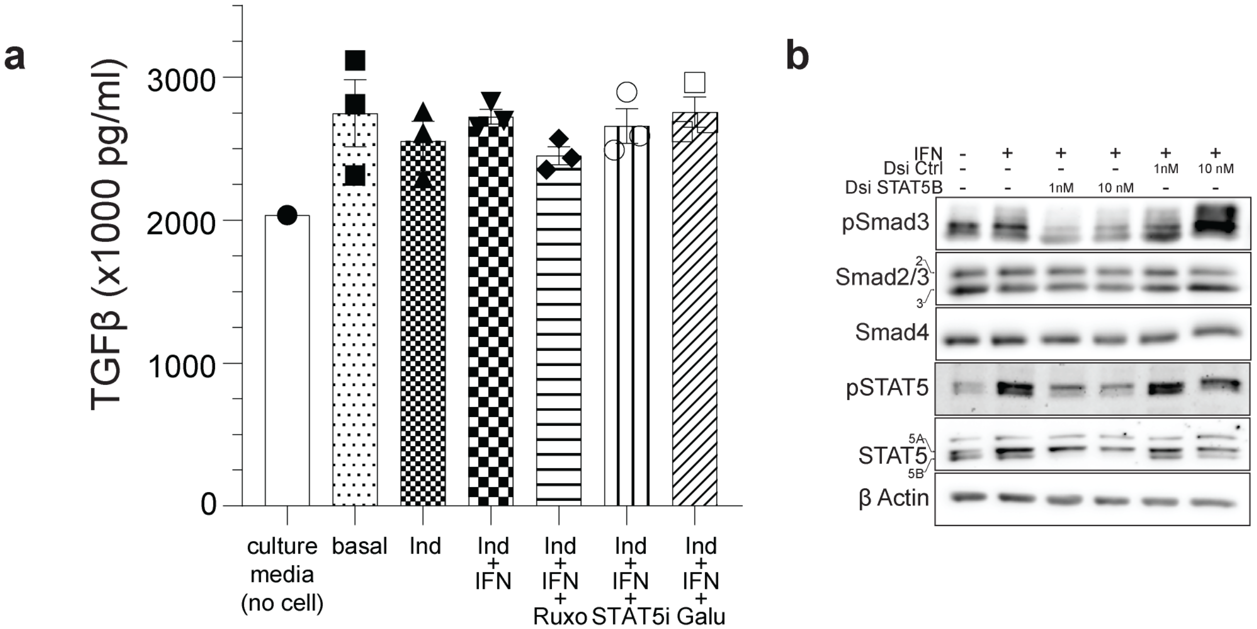

Supplementary Figure 3. Cross- regulation of IFNγ and TGFβ signaling pathways in hVA-MSC

(a) TGFβ measurement by sandwich ELISA of cell-culture supernatant after 5 days of adipogenic Induction with/out human IFNγ (10ng/ml) and/or specific antibodies as mentioned. Media was taken after 24 hours of exposure to cells. One way ANOVA and Tukey's post-test (not shown) indicates that values of any treatment condition is not significantly different than any other condition. (b) Western blot analysis of *STAT5B* knocked down hVA-MSCs after 7 days of treatment with/out IFNγ (10ng/ml).
Error bars represent mean ± SEM.

Supplementary figure 4

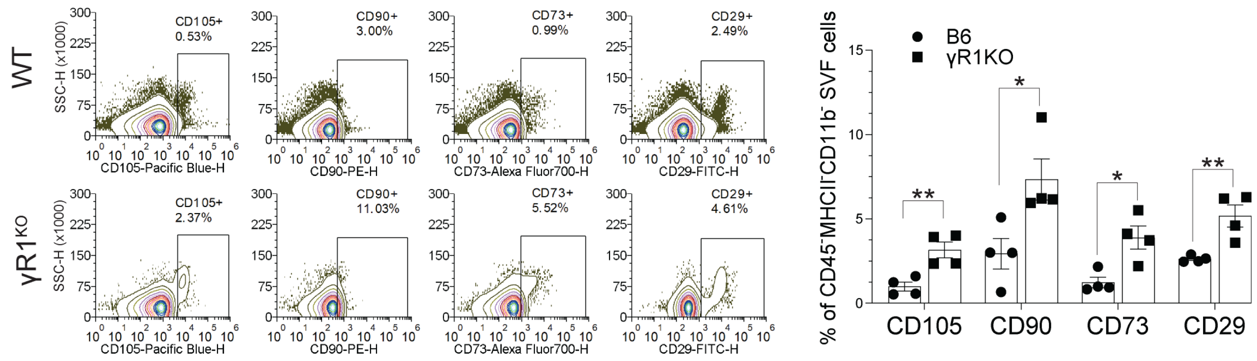

Supplementary Figure 4. γR1^{KO} mice shows increased VA-MSC population under chronic inflammatory conditions

Representative Phenotypic characterization (a) and quantification (b) of stromal vascular fraction (SVF) cells from HFD fed WT and γR1^{KO} epididymal fat pads using flow cytometry. Freshly isolated healthy SVF cells were selected using Ghost Red780 dye. Then CD45$^-$MHCII$^-$ CD11b$^-$ gated cells were probed for the expression of MSC specific markers (CD105+, CD90+, CD73+, CD29+). Gates are based on Fluorescent minus one (FMO) analysis. N=4 for (d). Error bars represent mean ± SEM. * indicates statistical significance (*$p < 0.05$; **$p < 0.005$) of Sidak's multiple comparison test followed by two way ANOVA.

Supplementary figure 5

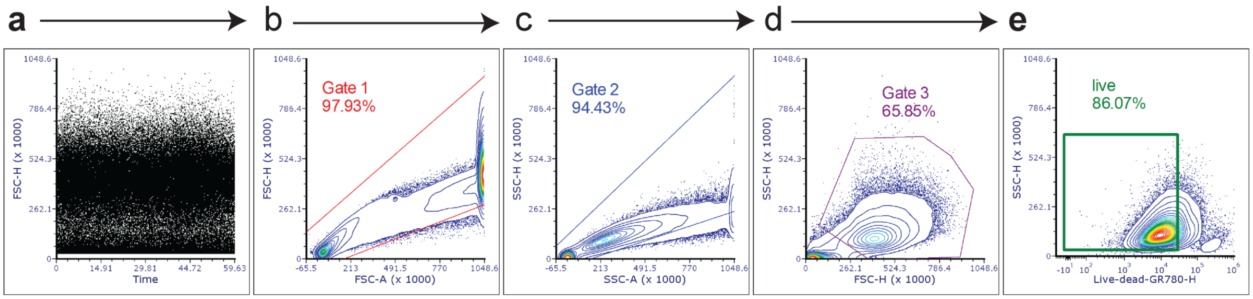

Supplementary Figure 5. General gating strategy for flow cytometry experiments used in this study

The cells are gradually refined to exclude debris, cell clumps and doublets from analysis using the following combination of gates-

(a) FSC-H/time to confirm uninterrupted, continuous flow of cells during data acquisition. (b) FSC-H/FSC-A (c) SSC-H/SSC-A (d) SSC-H/FSC-H (e) SSC-H/Live-dead. After e, SSC-H/ specific antibody was used as shown in respective figures.

The following section shows full length chemiluminescent and corresponding light micrograph images of western blots shown in the manuscript.

In addition to binding to specific epitopes, some antibodies also showed non-specific antigen binding at significantly different molecular weight positions. In those cases, boxes are drawn around correct molecular weight bands to indicate the specific portion displayed in main figures.

Full blot image and corresponding molecular weight marker of blot strips shown in Figure 1

PPARY (for Fig. 1e)

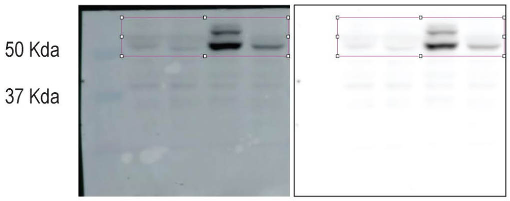

C/EBPα (for Fig. 1e)

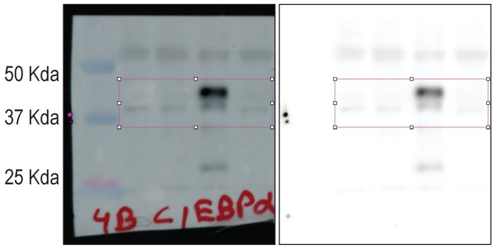

GAPDH (for Fig. 1e)

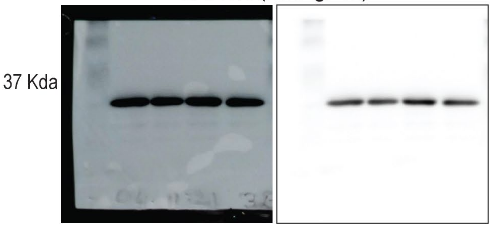

C/EBPδ (for Fig. 1g)

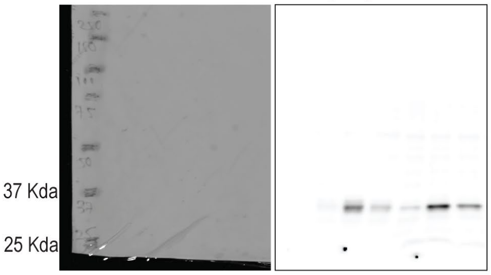

C/EBPβ (for Fig. 1g)

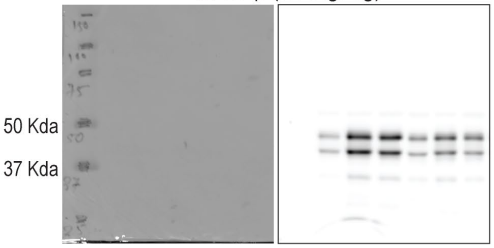

PPARα (for Fig. 1g)

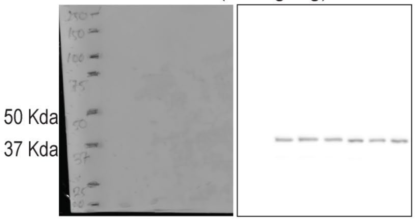

PPARY (for Fig. 1g)

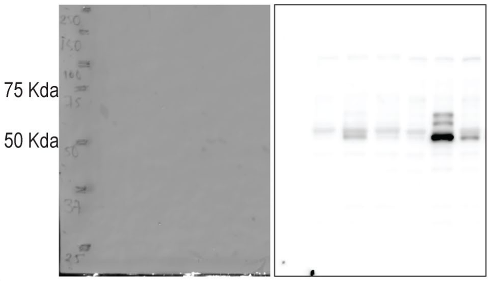

GAPDH (for Fig. 1g)

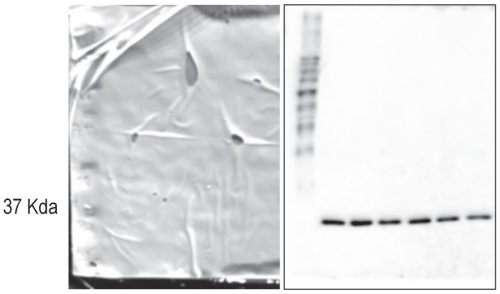

Full blot image and corresponding molecular weight marker of blot strips shown in Figure 2a

pSTAT1 (for Fig. 2a)

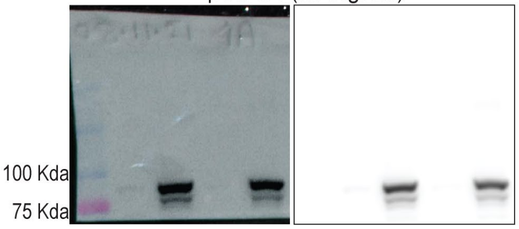

pSTAT3 (for Fig. 2a)

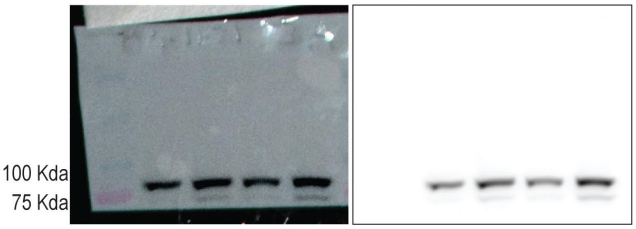

pSTAT5 (for Fig. 2a)

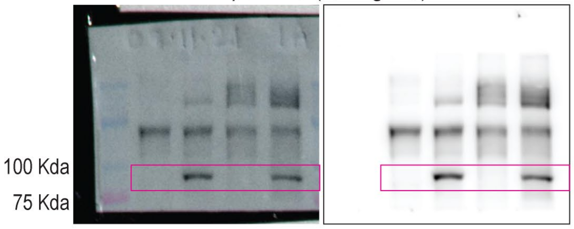

pJAK2 (for Fig. 2a)

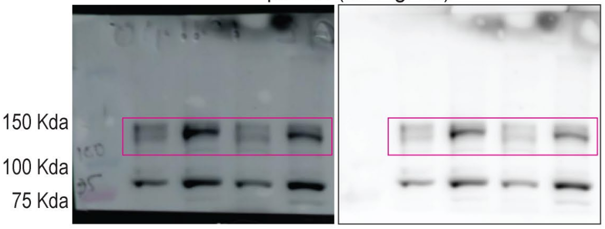

tSTAT1 (for Fig. 2a)

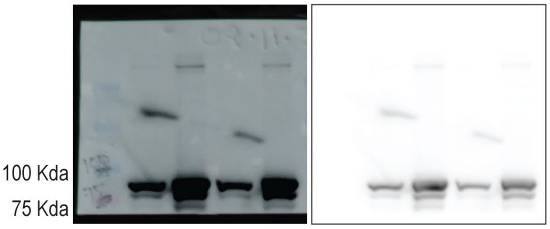

tSTAT3 (for Fig. 2a)

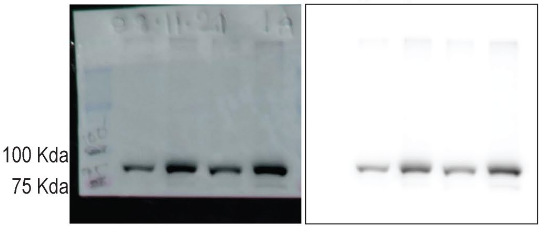

tSTAT5 (for Fig. 2a)

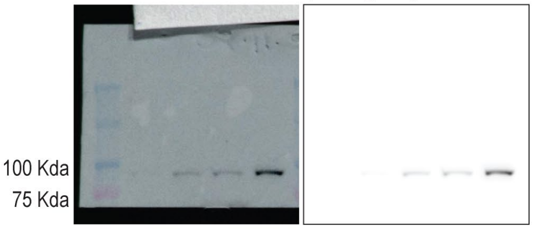

tJAK2 (for Fig. 2a)

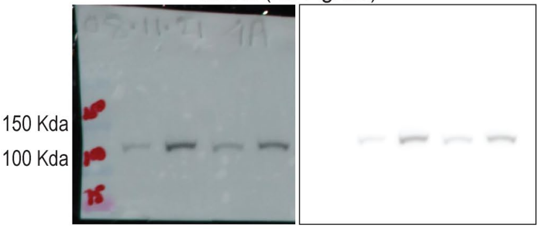

GAPDH (for Fig. 2a)

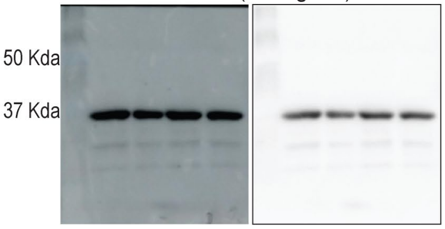

Full blot image and corresponding molecular weight marker of blot strips shown in Figure 2d

pSTAT1 (for Fig. 2d)

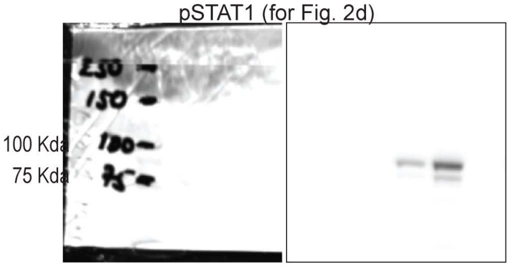

pSTAT3 (for Fig. 2d)

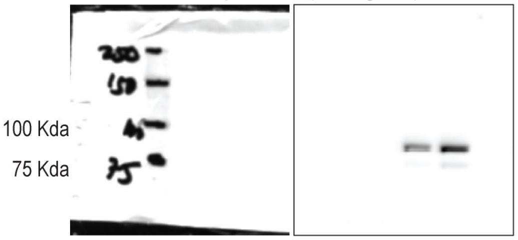

pSTAT5 (for Fig. 2d)

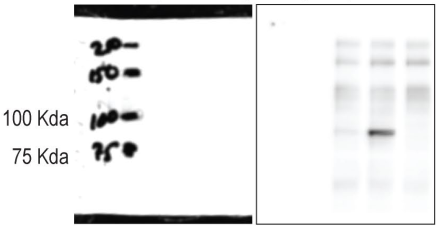

tSTAT1 (for Fig. 2d)

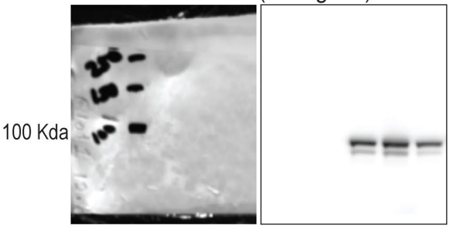

tSTAT3 (for Fig. 2d)

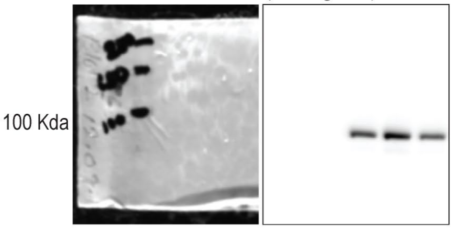

tSTAT5 (for Fig. 2d)

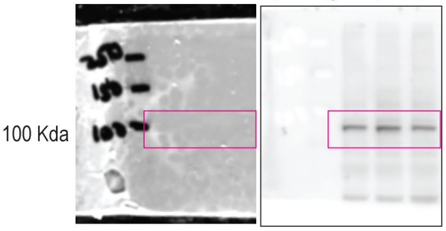

GAPDH (for Fig. 2d)

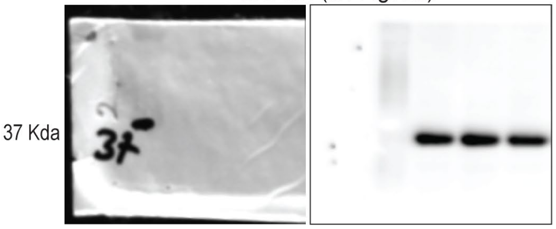

Full blot image and corresponding molecular weight marker of blot strips shown in Figure 3b

pSTAT1 (for Fig. 3b)

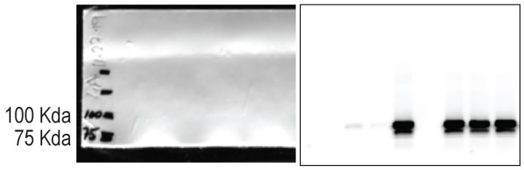

pSTAT3 (for Fig. 3b)

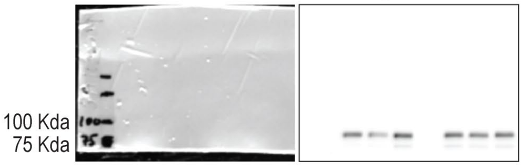

pSTAT5 (for Fig. 3b)

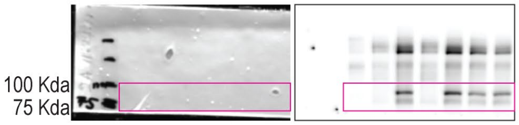

pJAK2 (for Fig. 3b)

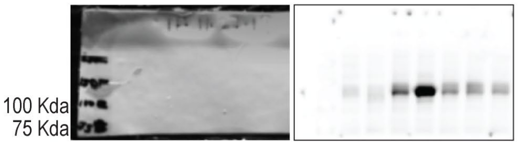

tSTAT1 (for Fig. 3b)

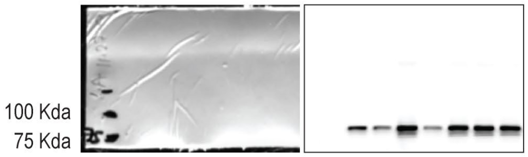

tSTAT3 (for Fig. 3b)

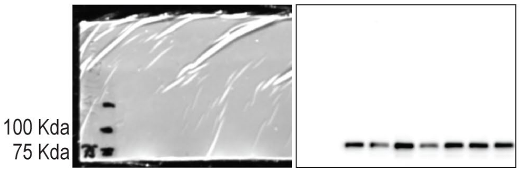

tSTAT5 (for Fig. 3b)

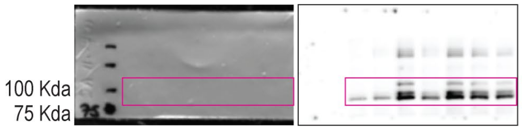

tJAK2 (for Fig. 3b)

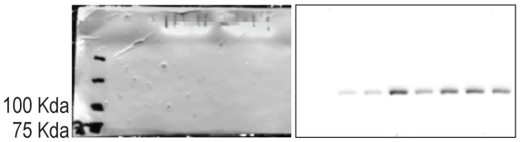

GAPDH (for Fig. 3b)

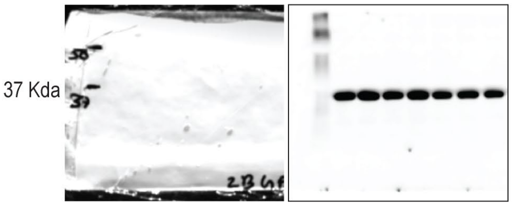

Full blot image and corresponding molecular weight marker of blot strips shown in Figure 3d

PPARY (for Fig. 3d)

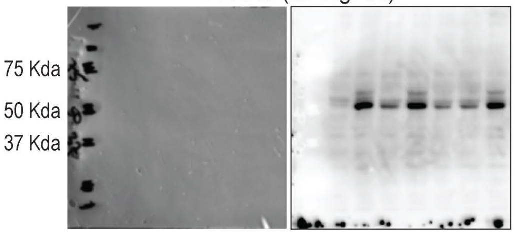

CEBPα (for Fig. 3d)

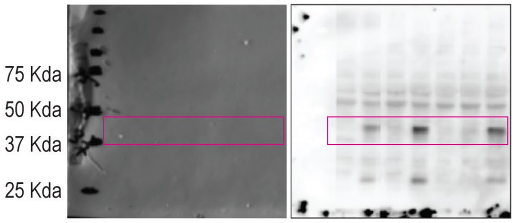

Perilipin (for Fig. 3d)

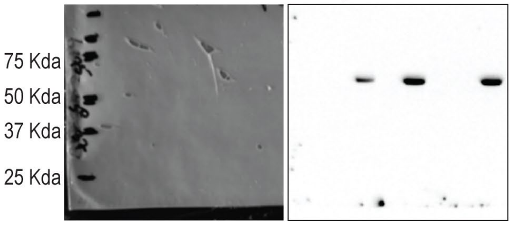

GAPDH (for Fig. 3d)

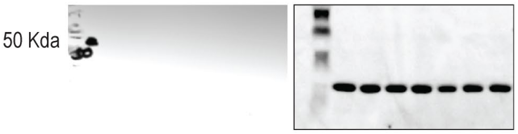

Full blot image and corresponding molecular weight marker of blot strips shown in Figure 4 a,b

PPARY (for Fig. 4a)

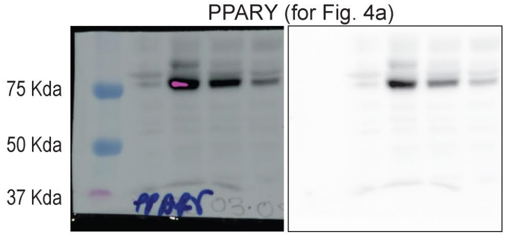

C/EBPα (for Fig. 4a)

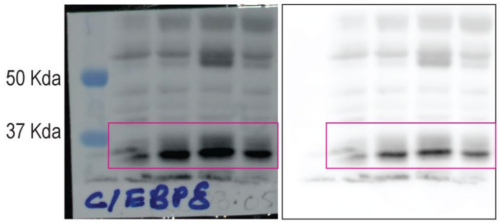

GAPDH (for Fig. 4a)

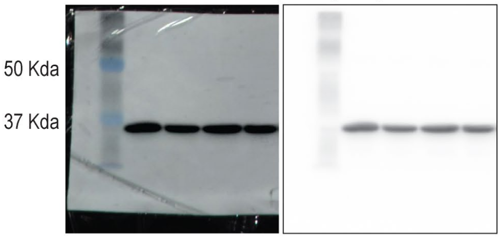

pSmad3 (for Fig. 4b)

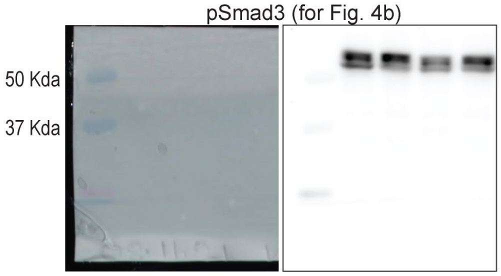

pSmad2 (for Fig. 4b)

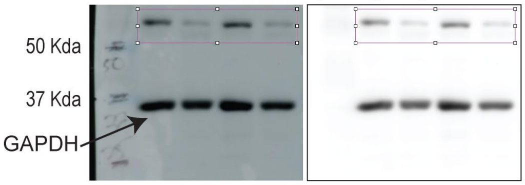

tSmad2/3 (for Fig. 4b)

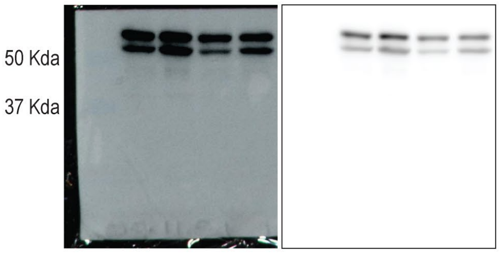

Smad4 (for Fig. 4b)

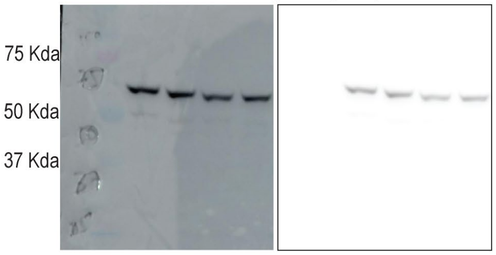

GAPDH (for Fig. 4b)

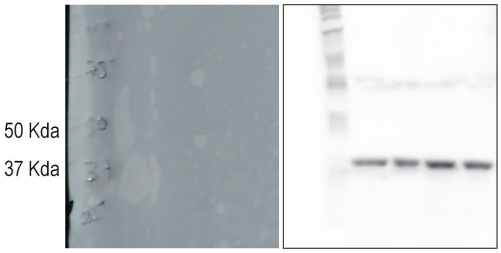

Full blot image and corresponding molecular weight marker of blot strips shown in Figure 4e
Note: lanes shown were not placed directly adjacent to molecular weight marker lane,
so boxes are drawn around the lanes to indicate their position.

pSmad3 (for Fig. 4e)

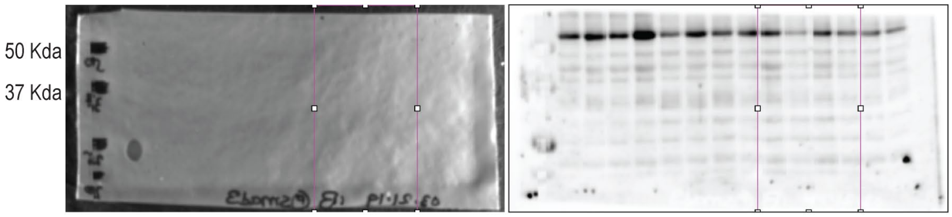

pSmad2 (for Fig. 4e)

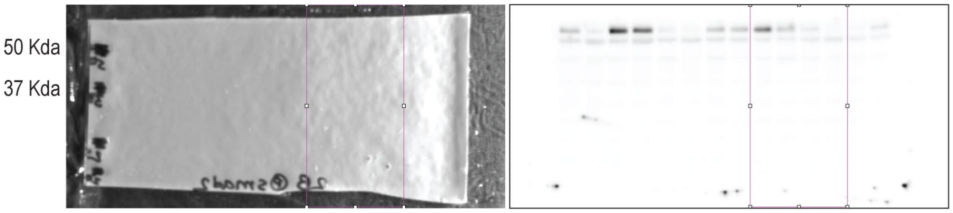

tSmad2/3 (for Fig. 4e)

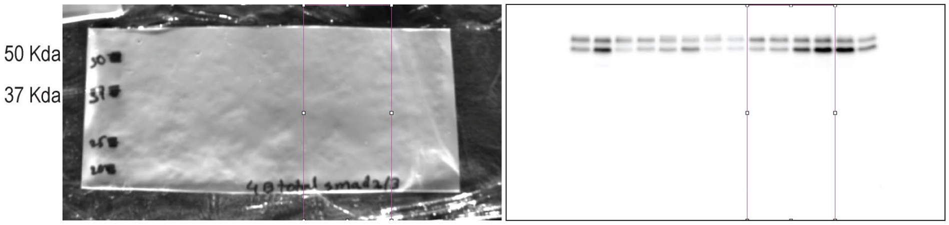

GAPDH (for Fig. 4e)

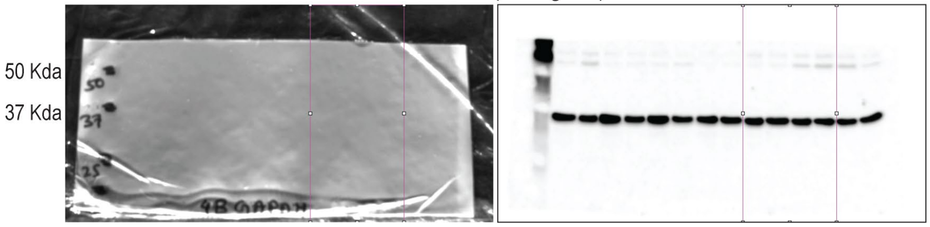

Full blot image and corresponding molecular weight marker of blot strips shown in Figure 4f,J.
Note: lanes shown were not placed directly adjacent to molecular weight marker lane,
so boxes are drawn around the lanes to indicate their position.

PPARY (for Fig. 4f)

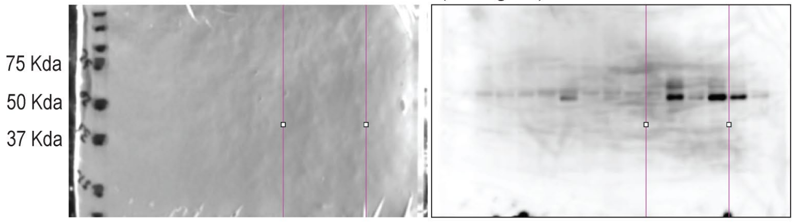

C/EBPα (for Fig. 4f)

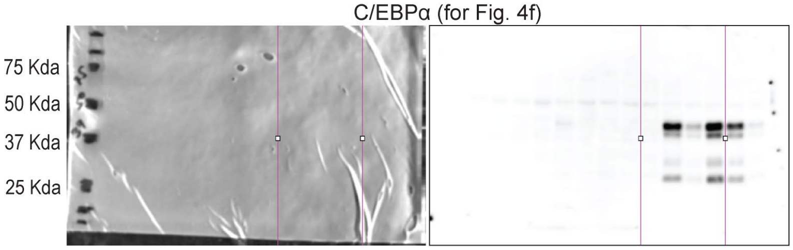

GAPDH (for Fig. 4f)

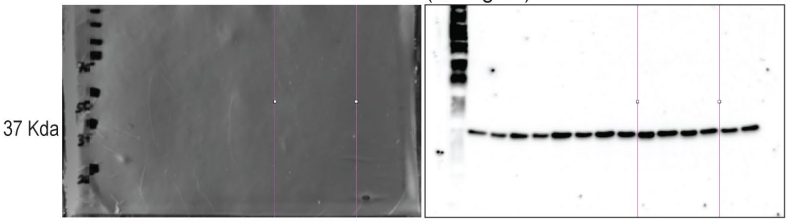

PPARY (for Fig. 4j)

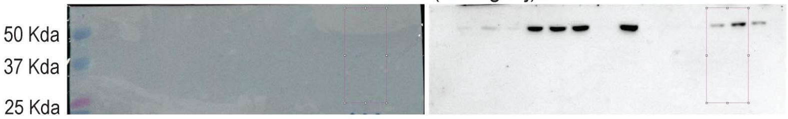

Smad2/3 (for Fig. 4j)

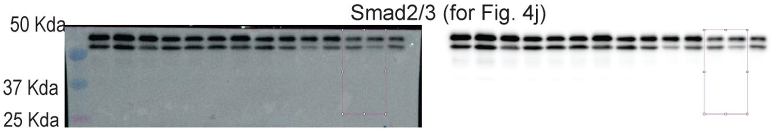

GAPDH (for Fig. 4j)

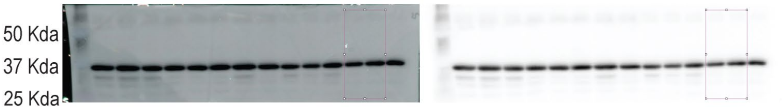

Full blot image and corresponding molecular weight marker of blot strips shown in Figure 5b.
Note: lanes shown were not placed directly adjacent to molecular weight marker lane,
so boxes are drawn around the lanes to indicate their position.

pSmad3 (for Fig. 5b)

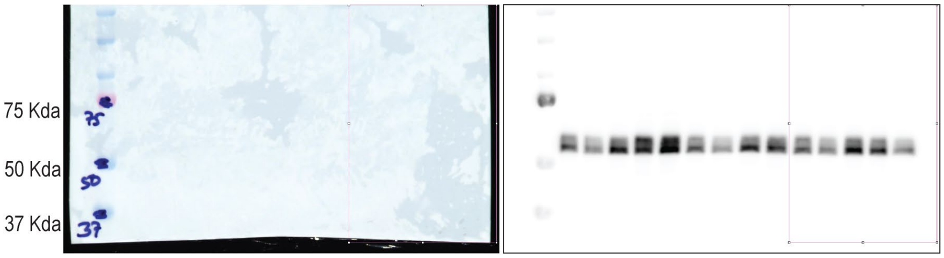

tSmad2/3 (for Fig. 5b)

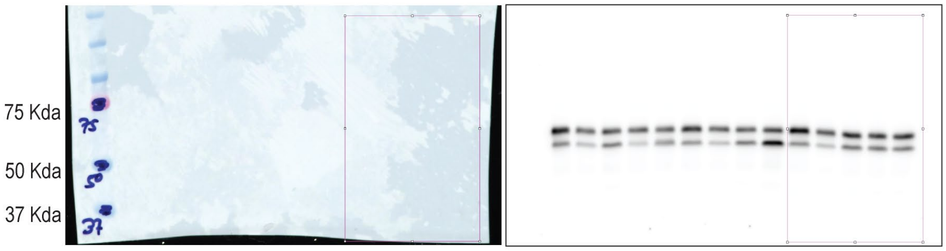

Smad4 (for Fig. 5b)

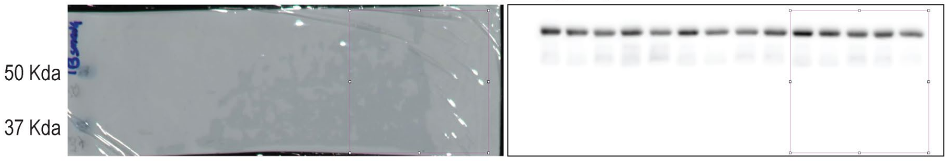

GAPDH (for Fig. 5b)

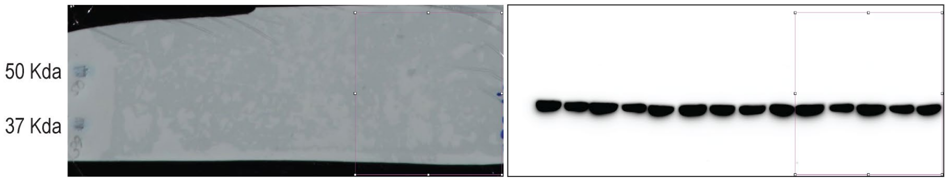

Full blot image and corresponding molecular weight marker of blot strips shown in Figure 5d. Note: lanes shown were not placed directly adjacent to molecular weight marker lane, so boxes are drawn around the lanes to indicate their position.

pStat5 (for Fig. 5d)

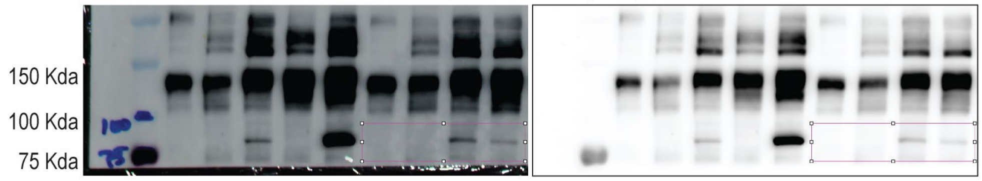

pStat1 (for Fig. 5d)

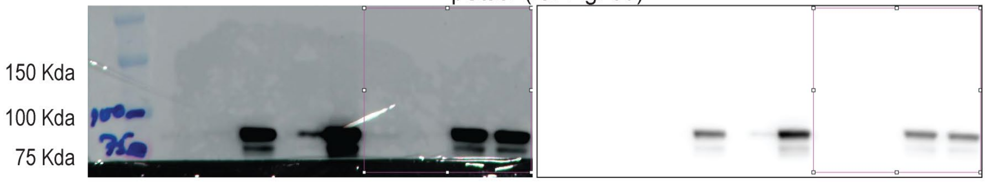

pStat3 (for Fig. 5d)

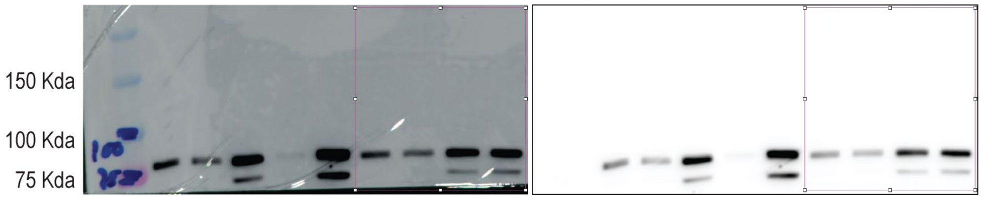

tStat5 (for Fig. 5d)

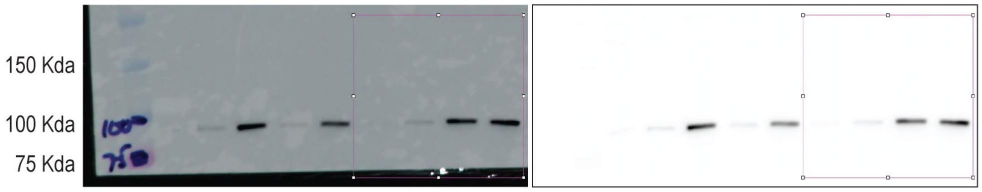

tStat3 (for Fig. 5d)

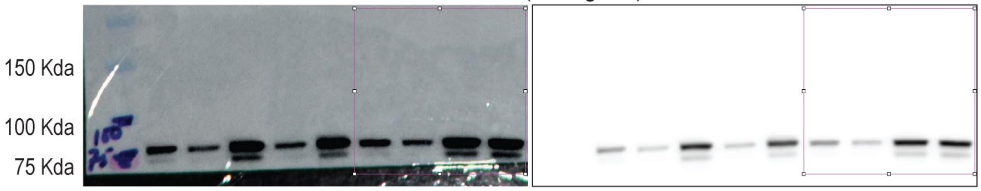

tStat1 (for Fig. 5d)

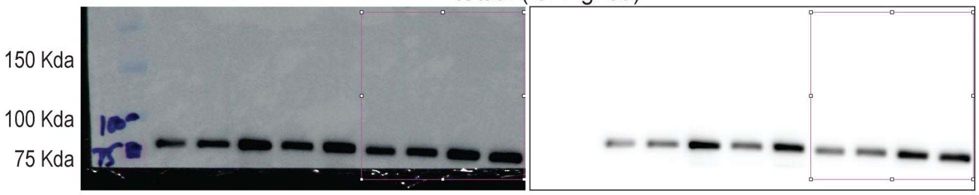

Actin (for Fig. 5d)

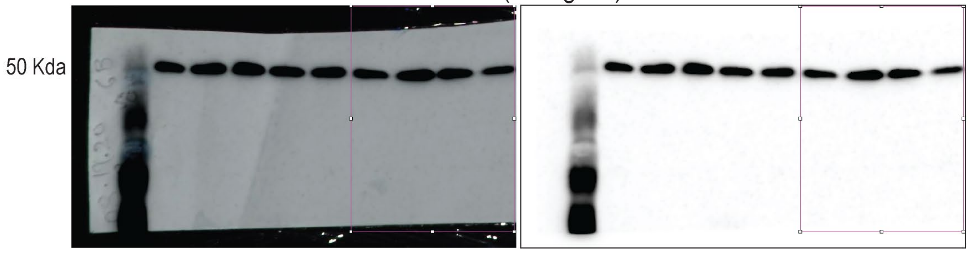

Full blot image and corresponding molecular weight marker of blot strips shown in Figure 5e. (lanes marked with box). Note: samples for Fig. 5b, 5d and 5e were ran on adjacent lanes of same gels.

pSmad3 (for Fig. 5e)

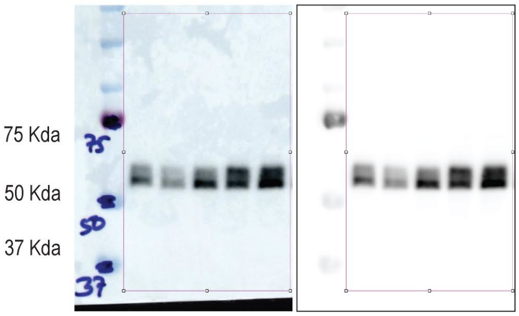

pSmad2 (for Fig. 5e)

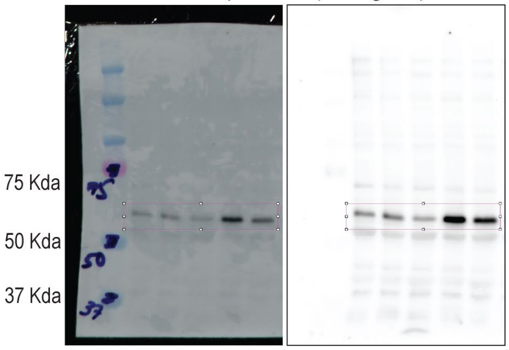

Smad4 (for Fig. 5e)

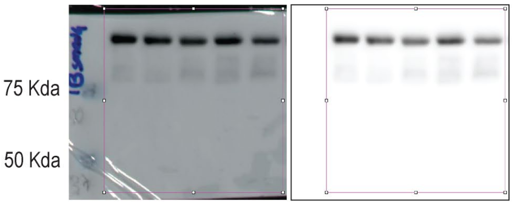

Smad7 (for Fig. 5e)

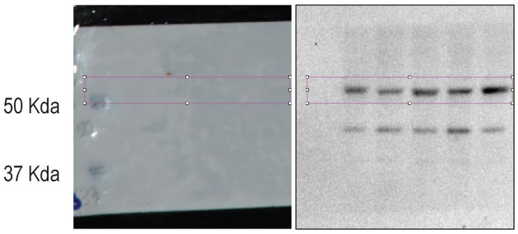

Smad2/3 (for Fig. 5e)

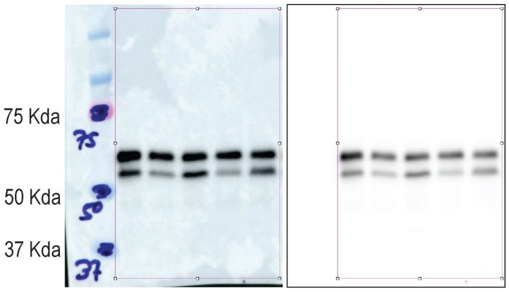

pSTAT1 (for Fig. 5e)

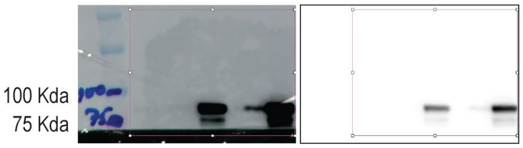

pSTAT3 (for Fig. 5e)

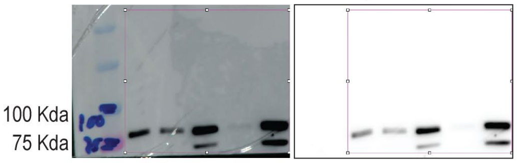

pSTAT5 (for Fig. 5e)

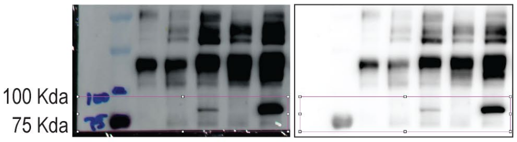

tSTAT1 (for Fig. 5e)

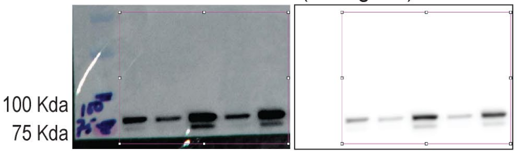

tSTAT3 (for Fig. 5e)

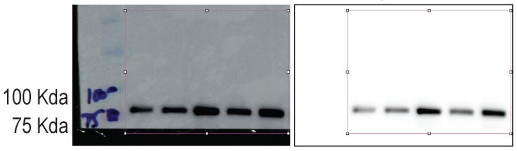

tSTAT5 (for Fig. 5e)

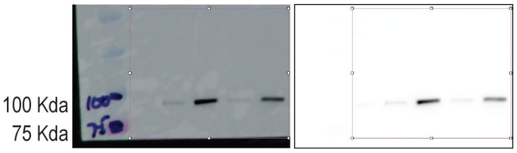

Actin (for Fig. 5e)

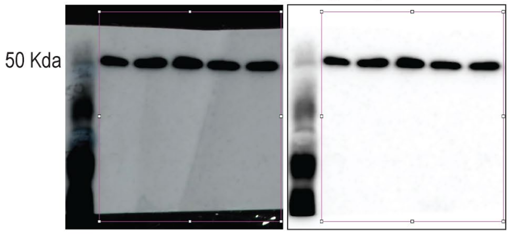

Full blot image and corresponding molecular weight marker of blot strips shown in Fig. 5h, 5i, and 5j.Note: 5h and 5i samples were ran on adjacent lanes of same gels. For 5j, lanes shown were not placed directly adjacent to molecular weight marker lane, so boxes are drawn around the lanes to indicate their position.

tSTAT5 (for Fig. 5h and 5i)

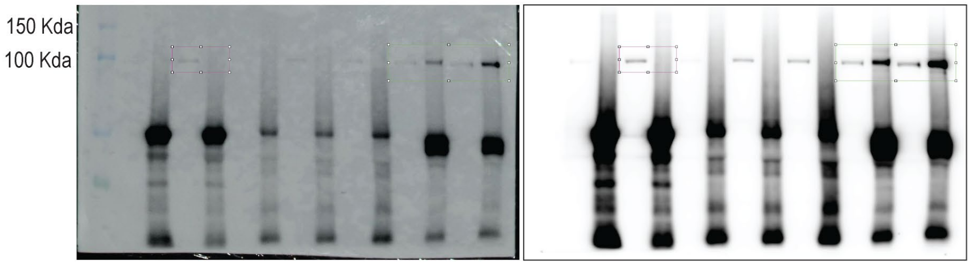

Smad2/3 (for Fig. 5h and 5i)

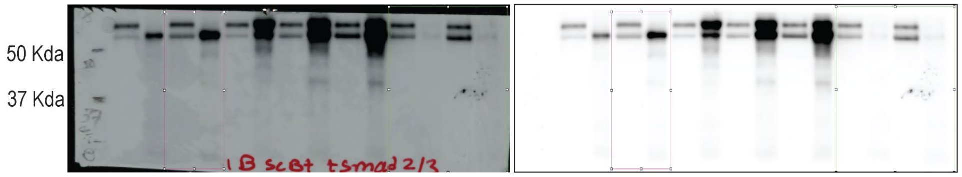

Smad4 (for Fig. 5h and 5i)

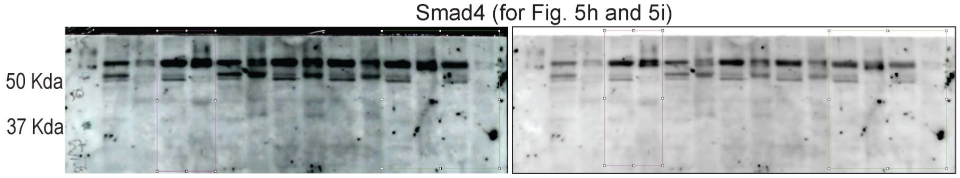

Smad1 (for Fig. 5h and 5i)

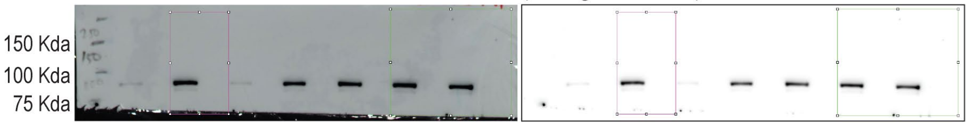

Smad3 (for Fig. 5h and 5i)

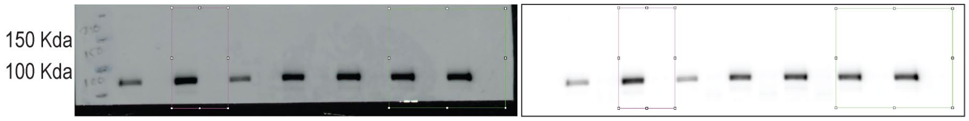

Smad4 (for Fig. 5j)

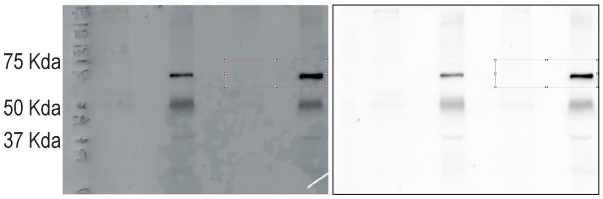

Smad2/3 (for Fig. 5j)

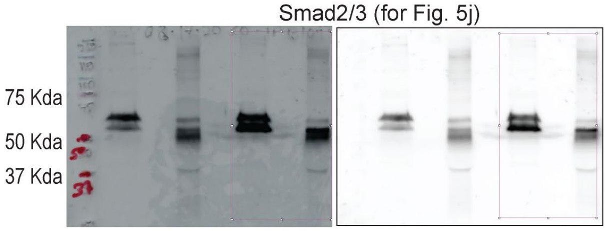

STAT5 (for Fig. 5j)

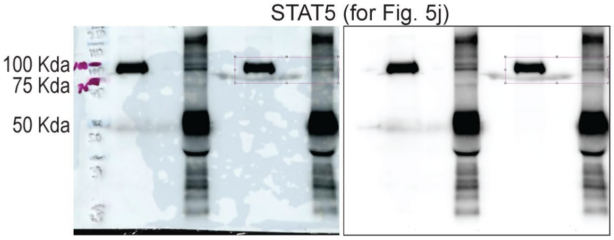

Full blot image and corresponding molecular weight marker of blot strips shown in Fig. 6d. Note: samples shown in Fig. 3d and 6d were ran on adjacent lanes of same gels. As 6d blot strips are not directly adjacent to molecular weight marker lane, boxes are drawn around the lanes shown in main figure.

PPARY(for Fig. 6d)

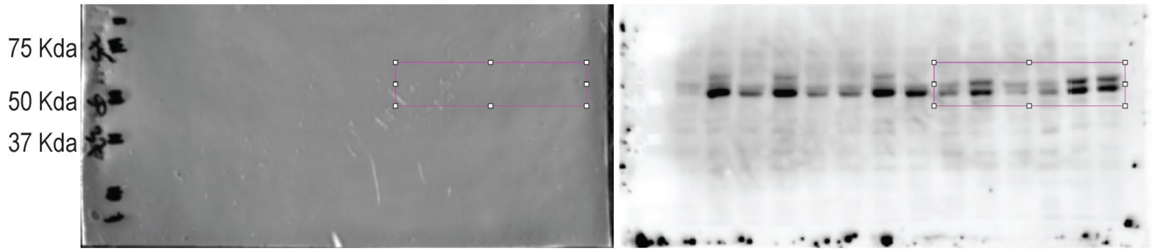

C/EBPα (for Fig. 6d)

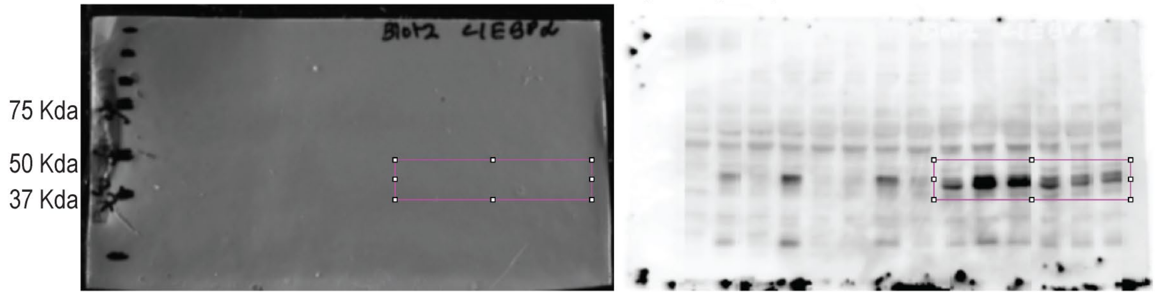

Perilipin (for Fig. 6d)

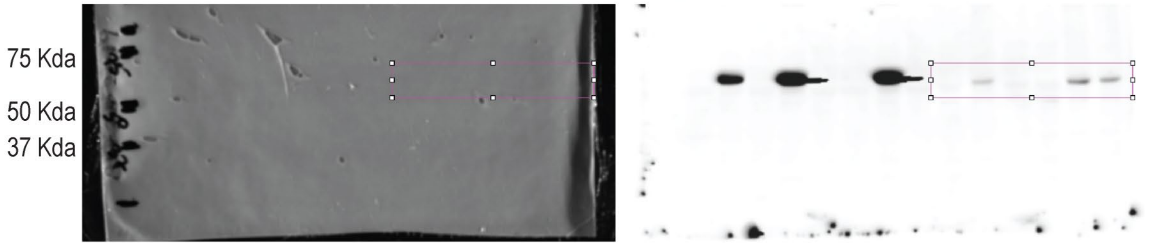

GAPDH (for Fig. 6d)

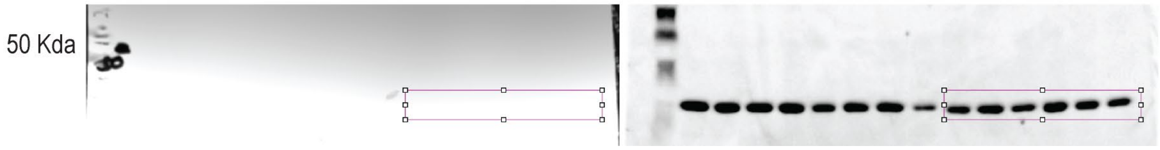

Supplementary tables

Table-1: Mouse visceral MSC adipogenic differentiation cocktail composition

**Reagent**	**Stock conc.**	**Stock solvent**	**Final conc.**
Dexamethasone	50mM	DMSO	4μM
Indomethicine	200mM	DMSO	31.25μM
IBMX	250mM	DMSO	1mM
Insulin	10mg/ml	-	5μg/ml

Table-2: Human visceral MSC adipogenic differentiation cocktail composition

**Reagent**	**Stock conc.**	**Stock solvent**	**Final conc.**
Dexamethasone	50mM	DMSO	8.5μM
Rosiglitazone	50mM	DMSO	8.5μM
IBMX	250mM	DMSO	160μM
Insulin	10mg/ml	-	10μg/ml

Table-3: Primer sequences for QRT PCR analysis

**Primer**	**Sequence (5'→ 3')**
hSTAT5 forward	GCCACCATCACGGACATTAT
hSTAT5 reverse	CAAACTTGGTCTGGGTCTTCA
hSTAT1 forward	GGTTCACTATAGTTGCGGAGAG

hSTAT1 reverse	GGGTCATGTTCGTAGGTGTATT
hSTAT3 forward	GAGAAGGACATCAGCGGTAAG
hSTAT3 reverse	CAGTGGAGACACCAGGATATTG
hJak2 forward	GAGCCTATCGGCATGGAATATC
hJak2 reverse	CCACTGAGCAAAGAGGTAAGAC
hPPARG forward	CTCAAACGAGAGTCAGCCTTTA
hPPARG reverse	GTGGGAGTGGTCTTCCATTAC
hCEBPD forward	CCTGGACTTACCACCACTAAAC
hCEBPD reverse	GCTGCATCAACAGGAGTAAGA
hCEBPa forward	GATAACCTTGTGCCTTGGAAATG
hCEBPa reverse	GAGGCAGGAAACCTCCAAATA
hGAPDH forward	GGTGTGAACCATGAGAAGTATGA
hGAPDH reverse	GAGTCCTTCCACGATACCAAAG

Table 4- Antibodies used

Antibody	Source	Catalog number
pJAK1	Cell Signaling Technology	Cat# 74129S
pJAK2	Cell Signaling Technology	Cat#66245S
pSTAT1	Cell Signaling Technology	Cat#9167S

pSTAT2	Cell Signaling Technology	Cat# 88410S
pSTAT3	Cell Signaling Technology	Cat# 9145S
pSTAT5	Cell Signaling Technology	Cat# 9359S
pSTAT6	Cell Signaling Technology	Cat# 56554S
pSmad2	Cell Signaling Technology	Cat# 18338S
PSmad3 (phospho S423 + S425)	Abcam	Cat# ab52903
STAT1	Cell Signaling Technology	Cat# 14994S
STAT3	Cell Signaling Technology	Cat# 12640S
STAT5	Cell Signaling Technology	Cat# 25656S
STAT6	Cell Signaling Technology	Cat# 56554S
Smad4 (note- used for human MSC samples)	Cell Signaling Technology	Cat# 46535S
Smad4 (note- used for mouse MSC samples)	Santa Cruz Biotechnology	Cat#sc-7966
perilipinx	Cell Signaling Technology	Cat# 9349S
PPARγ	Cell Signaling Technology	Cat# 2435S
C/EBPα	Cell Signaling Technology	Cat# 8178S
B-Actin	Cell Signaling Technology	Cat# 4970S
PPARα	Santa Cruz Biotechnology	Cat# sc-130640
Smad3	Cell Signaling Technology	Cat# 9523S
Smad2/3 Antibody (C-8)	Santa Cruz Biotechnology	Cat# sc-133098
Smad7	Santa Cruz Biotechnology	Cat# sc-365846
GAPDH	Santa Cruz Biotechnology	Cat# sc-137179
C/EBPδ	Santa Cruz Biotechnology	Cat# sc-365546
C/EBPβ	Santa Cruz Biotechnology	Cat# sc-7962
TGFBR1 (note- used for Flow cytometry)	Santa Cruz Biotechnology	Cat# sc-518018
Mouse IgG2ak Isotype Control for TGFBR1	Thermo-FIscher	Cat#14-4724-82

α- human CD54 APC	Miltenyl Biotech	130-104-216
Isotype control for above	Miltenyl Biotech	130-113-446
α- human CD105 PE	BD Biosciences	Cat#560839
Mouse IgG1k Isotype Control for above	BD Biosciences	Cat#554680
α- human CD90 APC	BD Biosciences	Cat#559869
Mouse IgG1k Isotype Control for above	BD Biosciences	Cat#555751
α- human CD73 PE	BD Biosciences	Cat#550257
Mouse IgG1k Isotype Control for above	BD Biosciences	Cat#556027
α- human CD45 FITC	BD Biosciences	Cat#555482
Mouse IgG1k Isotype Control for above	BD Biosciences	Cat#555748
α- human CD11b Pacific Blue	BD Biosciences	Cat#558123
Mouse IgG1k Isotype Control for above	BD Biosciences	Cat#558120
α- human CD34 PE	Miltenyi Bio	Cat#130-113-179
Mouse IgG2k Isotype Control for above	BD Biosciences	Cat#553475
α- human HLA-DR PerCp	BD Biosciences	Cat#347364
Mouse IgG2k Isotype Control for above	BD Biosciences	Cat#340765
α- mouse CD105 FITC	Invitrogen	Cat#MA5-17945
Rat IgG2ak isotype control for above	BD Biosciences	Cat#553929
α- mouse CD29 FITC	Biolegend	Cat#102025
Hamster IgG2k isotype control for above	BD Biosciences	Cat#553964
α- mouse CD44 PE	BD Biosciences	Cat#553134
α- mouse CD11b PE	invitrogen	Cat#12-0112-82
α- mouse CD45 PE	BD Biosciences	Cat#553081
Rat IgG2bk isotype control for CD44, Cd11b, CD45	BD Biosciences	Cat#553989

α- mouse CD73 PE	BD Biosciences	Cat#550741
Rat IgG2ak isotype control for above	eBiosciences	Cat#12-4321-81
α- mouse Ly6A/E PECy7	BD Biosciences	Cat#558162
Rat IgG2ak isotype control for above	BD Biosciences	Cat#552784
α- mouse I-A|b| (MHCII) PE	BD Biosciences	Cat#553552
Mouse IgG2ak isotype control for above	BD Biosciences	Cat#553457
α- mouse CD105 (pacific Blue)	Biolegend	Cat#120412
α- mouse CD73 (Alexa Fluor 700)	Biolegend	Cat#127230
α- mouse CD45 (BV570)	Biolegend	Cat#103136
α- mouse I-AB (APC)	Biolegend	Cat#116417
α- mouse CD90 (PE)	Biolegend	Cat#105307
α- mouse CD11b (PE-Cy5)	Biolegend	Cat#101209
Rabbit anti-Goat IgG (H+L) Cross-Adsorbed Secondary Antibody, Alexa Fluor 660	ThermoFisher	Cat# A-21073
Goat anti-Rabbit IgG (H+L) Cross-Adsorbed Secondary Antibody, Alexa Fluor 594	ThermoFisher	Cat# A-11080
Goat anti-Mouse IgG (H+L)/Alexa Fluor plus 488	ThermoFisher	Cat# A32723
Goat anti-Rabbit IgG (H+L) , Alexa Fluor Plus 555	ThermoFisher	Cat# A32732
Rat anti-Mouse IgG1 Secondary Antibody, APC	Thermo-FIscher	Cat#17-4015-82

Table 5- reagents list

Reagent	Source	Catalog number
Thermo Scientific™ Triglycerides Reagent	Fischer Scientific	Cat# TR22421
Triglyceride Mix	Sigma Aldrich	Cat# 17810-1AMP-S
Oil Red O solution	Sigma Aldrich	Cat# O1391
Hematoxylin Solution, Harris Modified	Sigma Aldrich	Cat #HHS16
Galunisertib (LY2157299)	Selleckchem	Cat# S2230
Insulin	Sigma Aldrich	Cat #I0516
dexamethasone	Cayman	Cat# 11015
rosiglitazone	Cayman	Cat# 71740
IBMX	Cayman	Cat# 13347
Indomethacin	Cayman	Cat# 70270
Fludarabine	Targetmol	Cat# T1038
Static	Targetmol	Cat# T6308
Ruxolitinib	Targetmol	Cat# T3043
Stat5i	Targetmol	Cat #T4216
Galunisertib (LY2157299)	Selleckchem	Cat# S2230
SIS3	Cayman Chemicals	Cat# 15945
MG132	Targetmol	Cat#T2154
Alexa Fluor™ 647 Phalloidin	ThermoFisher	Cat# A22287
Acti-stain 555 phalloidin	FisherScientific	Cat# 50-646-254
Acti-stain 488 phalloidin	Fisher/cytoskeleton.inc	Cat# PHDG1-A
Pierce™ Protein A/G Magnetic Beads	ThermoFisher	Cat#88802
Normal goat serum 100ml	ThermoFisher	Cat# 50062Z

Recombinant Murine IFN-γ	Peprotech	Cat# 315-05
Recombinant Human IFN-γ	Peprotech	Cat# 300-02
Recombinant Human EGF	Peprotech	Cat# 100-15
Recombinant Human GM-CSF	Peprotech	Cat# 300-03
Recombinant Human IL6	Peprotech	Cat# 200-06
Recombinant Human IL1B	Peprotech	Cat# 200-01B
Recombinant Human PDGF	Peprotech	Cat# 100-00AB
Recombinant Mouse TGF-β1	Biolegend	Cat# 763102
Recombinant Human TNFα	R&D	Cat# 10291-TA-050
Recombinant Human IL15	R&D	Cat# 247-ILB-025/CF
Recombinant Human IL4	R&D	Cat# 204-IL-020/CF
Recombinant Human Ccl2	R&D	Cat# 279-MC
Recombinant Human TGF-β1	R&D	Cat# 7754-BH-005
Recombinant Human IL7	R&D	Cat# 207-IL
Recombinant Human IL2	R&D	Cat# 202-IL
Molecular Probes™ ProLong™ Diamond Antifade Mountant	FisherScientific	Cat# P36970
Ultra Sensitive Mouse Insulin ELISA Kit	Crystal Chem	Cat# 90080
Mouse IFN-γ ELISA	eBiosciences	Cat#14-7313-688
Human/mouse TGF-β ELISA	eBiosciences	Cat#14-8352-68
RNeasy Mini kit	Qiagen	Cat#74106
QuantiTect Reverse Transcription Kit	Qiagen	Cat#205311
QuantiTect SYBR® Green PCR Kits	Qiagen	Cat#204143

Table-6: Sequences for dSi RNAs

Dsi RNA	Sequence
STAT5B 13.1 forward (5'-3')	UGGGAGACUUGAAUUACCUUAUCTA
STAT5B 13.1 reverse (3'-5')	UAGAUAAGGUAAUUCAAGUCUCCCAAG
SMAD3 13.1 forward (5'-3')	AGUCAGUUGCAUUCAUUAAAUCAAC
SMAD3 13.1 reverse (3'-5')	GUUGAUUUAAUGAAUGCAACUGACUAC
STAT1 13.1 forward (5'-3')	CUUGACAGUAAAGUCAGAAAUGUGA
STAT1 13.1 reverse (3'-5')	UCACAUUUCUGACUUUACUGUCAAGCU
