# Supplementary material for: A STAT5-Smad3 dyad regulates adipogenic plasticity of visceral adipose mesenchymal stromal cells during chronic inflammation
Source: NPJ Regen Med. 2022 Aug 31;7:41. doi: 10.1038/s41536-022-00244-5 (PMC9433418; doi:10.1038/s41536-022-00244-5)
Supplement: Supplementary file 2 — REPORTING SUMMARY [file 41536_2022_244_MOESM2_ESM.pdf]

nature portfolio

Corresponding author(s): Dr Jacques Galipeau

Last updated by author(s): Jul 26, 2022

Reporting Summary

Nature Portfolio wishes to improve the reproducibility of the work that we publish. This form provides structure for consistency and transparency in reporting. For further information on Nature Portfolio policies, see our Editorial Policies and the Editorial Policy Checklist.

Statistics

For all statistical analyses, confirm that the following items are present in the figure legend, table legend, main text, or Methods section.

n/a	Confirmed	
☐	☒	The exact sample size (n) for each experimental group/condition, given as a discrete number and unit of measurement
☐	☒	A statement on whether measurements were taken from distinct samples or whether the same sample was measured repeatedly
☐	☒	The statistical test(s) used AND whether they are one- or two-sided<br>*Only common tests should be described solely by name; describe more complex techniques in the Methods section.*
☐	☒	A description of all covariates tested
☐	☒	A description of any assumptions or corrections, such as tests of normality and adjustment for multiple comparisons
☒	☐	A full description of the statistical parameters including central tendency (e.g. means) or other basic estimates (e.g. regression coefficient) AND variation (e.g. standard deviation) or associated estimates of uncertainty (e.g. confidence intervals)
☒	☐	For null hypothesis testing, the test statistic (e.g. F, t, r) with confidence intervals, effect sizes, degrees of freedom and P value noted<br>*Give P values as exact values whenever suitable.*
☒	☐	For Bayesian analysis, information on the choice of priors and Markov chain Monte Carlo settings
☒	☐	For hierarchical and complex designs, identification of the appropriate level for tests and full reporting of outcomes
☒	☐	Estimates of effect sizes (e.g. Cohen's d, Pearson's r), indicating how they were calculated

Our web collection on statistics for biologists contains articles on many of the points above.

Software and code

Policy information about availability of computer code

Data collection	no novel software or code was used
Data analysis	No commercial, open sourse or custom code was used for data analysis. Only standard, publicly available softwares including NIH-ImageJ (V1.53q), GraphPad Prism (V9), NIS elements, FCS express (V7), LiCor Image Studio Lite (V5), Zeiss ZEN (V3), Adiposoft plugin in ImageJ, were used.

For manuscripts utilizing custom algorithms or software that are central to the research but not yet described in published literature, software must be made available to editors and reviewers. We strongly encourage code deposition in a community repository (e.g. GitHub). See the Nature Portfolio guidelines for submitting code & software for further information.

Data

Policy information about availability of data

All manuscripts must include a data availability statement. This statement should provide the following information, where applicable:

- Accession codes, unique identifiers, or web links for publicly available datasets
- A description of any restrictions on data availability
- For clinical datasets or third party data, please ensure that the statement adheres to our policy

The datasets generated during and/or analysed during the current study are available from the corresponding author on reasonable request.

Human research participants

Policy information about studies involving human research participants and Sex and Gender in Research.

Reporting on sex and gender	N/A
Population characteristics	N/A
Recruitment	N/A
Ethics oversight	N/A

Note that full information on the approval of the study protocol must also be provided in the manuscript.

Field-specific reporting

Please select the one below that is the best fit for your research. If you are not sure, read the appropriate sections before making your selection.

[x] Life sciences   [ ] Behavioural & social sciences   [ ] Ecological, evolutionary & environmental sciences

For a reference copy of the document with all sections, see nature.com/documents/nr-reporting-summary-flat.pdf

Life sciences study design

All studies must disclose on these points even when the disclosure is negative.

Sample size	The number of mice used in this study was based on published articles in the field focusing on similar phenotypic analysis. For one recent example, please see "β-Cell Knockout of SENP1 Reduces Responses to Incretins and Worsens Oral Glucose Tolerance in High-Fat Diet–Fed Mice- Lin et. al., Diabetes 2021;70(11):2626–2638", where similar sample size was used.<br>For primary human MSCs, preliminary adipogenesis related molecular biology analyses showed that prominent, statistically significant difference was achievable between control and treatment conditions using 3 distinct samples. Therefore, we used 3/4 such samples throughout this study.
Data exclusions	No data were excluded from analysis
Replication	human MSC samples from distinct donors were tested in an identical manner using same source/ batch of reagents as much as possible. For animal studies, animals were kept in same room in cages next to each other and were treated identically, including giving same batch and same quantitiy of food. All attempts at replication were successful, as revealed by statistical analysis.
Randomization	Randomization is not relevant to this study since (a) animals of different genotypes were compared and (b) all human MSC samples were obtained from healthy donors; these MSC samples were treated differently according to the experimental designs.
Blinding	For all animal data, investigators were blinded to group allocation for data collection.<br>For all microscopy, colorimetric assays, flow cytometry, and qRT PCR analyses, investigators were blinded during data collection.<br>For western blots, blinding was not possible because relative position of samples on gels/blots were needed to be predetermined for presentation on manuscript figures. However, all blots were probed for housekeeping protein (GAPDH or β- Actin) to ensure equal loading.

Reporting for specific materials, systems and methods

We require information from authors about some types of materials, experimental systems and methods used in many studies. Here, indicate whether each material, system or method listed is relevant to your study. If you are not sure if a list item applies to your research, read the appropriate section before selecting a response.

Materials & experimental systems

n/a	Involved in the study	
[ ]	[x]	Antibodies
[x]	[ ]	Eukaryotic cell lines
[x]	[ ]	Palaeontology and archaeology
[ ]	[x]	Animals and other organisms
[x]	[ ]	Clinical data
[x]	[ ]	Dual use research of concern

Methods

n/a	Involved in the study	
[x]	[ ]	ChIP-seq
[ ]	[x]	Flow cytometry
[x]	[ ]	MRI-based neuroimaging

Antibodies

Antibodies used	A large number of antibodies were used in the study. Supplementary table section include the required details.
Validation	Only well described and previously cited antibodies were used from commercial vendors. Additionally, all antibodies were further validated in manuscript figures using negative controls (i.e. non- treated or inhibitor treated cells, gene knocked -down cells). For example, the specificity of the pSTAT5 and tSTAT5 antibody is shown by treating cells with STAT5i inhibitor which shows reduced band intensity in Fig.3. Similarly, the specificity of the PPAR gamma antibody was validated by probing adipogenically induced MSCs which showed prominent bands at expected molecular weight and no such band in non-induced cells.

Animals and other research organisms

Policy information about studies involving animals; ARRIVE guidelines recommended for reporting animal research, and Sex and Gender in Research

Laboratory animals	Species- Mouse (Mus musculus).<br>Strain- C57BL/6J (B6, stock no.000664) and B6.129S7-Ifngr1tm1Agt/J (γR1-KO, stock no. 003288).<br>Sex- Male animals.<br>Age- Age matched, ~30 weeks old animals which were fed high- fat diet for ~25 weeks were used.
Wild animals	This study did not involve wild animals.
Reporting on sex	These findings apply to male animals; as MSCs from male animals were used for in-vitro experiments and in vivo studies shown in Fig. 6 were done using male animals only (mentioned in figure legend). We initially collected adipose MSCs from both male and female animals. However, regardless of genotype, male MSCs culture-adopted much better and showed robust adipogenesis compared to female MSCs. Therefore, we continued with male MSCs and subsequently performed in vivo experiments using male animals.
Field-collected samples	This study did not involve field-collected samples.
Ethics oversight	Animal Care and Use Policies of the University of Wisconsin-Madison (IACUC ID - M006496).<br>University of Wisconsin-Madison Institutional Review Board (IRB ID- 2016-1545).

Note that full information on the approval of the study protocol must also be provided in the manuscript.

Flow Cytometry

Plots

Confirm that:

☒ The axis labels state the marker and fluorochrome used (e.g. CD4-FITC).

☒ The axis scales are clearly visible. Include numbers along axes only for bottom left plot of group (a 'group' is an analysis of identical markers).

☒ All plots are contour plots with outliers or pseudocolor plots.

☒ A numerical value for number of cells or percentage (with statistics) is provided.

Methodology

Sample preparation	MSC cells were obtained from human or mouse adipose tissue. After culturing in vitro with specific treatment conditions as required, flow cytometry analysis was done. For this, cells were detached from plate using accutase treatment and washed once with PBS. Cells were then washed twice in FACS buffer (PBS+5%FBS+0.09% sodium azide). Then, cells were resuspended in 100µl FACS buffer and incubated with primary antibodies for 30 minutes at 4°c. Cells were then washed twice again in FACS buffer, resuspended in 500 µl FACS buffer and analyzed using Attune Nxt flow cytometer. Live-dead staining was done with either Ghost Red 780 dye or DAPI according to the manufacturer recommendation. Data are presented as contour plots with outliers for supplementary figure 4. Fig. 4a was changed from contour to histogram plot based on reviewer's suggestion.For human and mouse MSC characterization (1a, 6a-b), histogram figures are presented for better comparison in regard to isotype control. Representative images (N= at least 3) are shown in figures.
Instrument	Attune NxT Acoustic focusing cytometer; model BRVx
Software	Data collection- Attune Cytometer software. Data analysis- FCS express V7
Cell population abundance	FACS Sorting/ post sorting analysis was not done in this study. For flow cytometry analysis, at least 20,000 post- live-dead gated ells were used per condition. About equal number of cells were used in each individual experiment.
Gating strategy	FSC-H/FSC-A --> SSC-H/SSC-A --> SSC-H/FSC-H --> SSC-H/Live-Dead --> SSC-H/specific antibody.<br>a figure exemplifying the gating strategy is provided in the Supplementary Figure 5.

☒ Tick this box to confirm that a figure exemplifying the gating strategy is provided in the Supplementary Information.
